# Pathogenic Differences of Type 1 Restriction-Modification Allele Variants in Experimental *Listeria monocytogenes* Meningitis

**DOI:** 10.3389/fcimb.2020.590657

**Published:** 2020-10-30

**Authors:** Florian R. Zbinden, Megan De Ste Croix, Denis Grandgirard, Richard D. Haigh, Irene Vacca, Roxana Zamudio, Emily C. A. Goodall, Roger Stephan, Marco R. Oggioni, Stephen L. Leib

**Affiliations:** ^1^Neuroinfection Laboratory, Institute for Infectious Diseases, University of Bern, Bern, Switzerland; ^2^Department of Genetics and Genome Biology, University of Leicester, Leicester, United Kingdom; ^3^Institute for Food Safety and Hygiene, Vetsuisse Faculty, University of Zurich, Zurich, Switzerland

**Keywords:** listeria monocytogenes (*L. monocytogenes*), meningoencephalitis, restriction modification systems, pathogenesis, inflammation, brain damage, neurolisteriosis

## Abstract

**Background:** *L. monocytogenes* meningoencephalitis has a mortality rate of up to 50% and neurofunctional sequelae are common. Type I restriction-modification systems (RMS) are capable of adding methyl groups to the host genome. Some contain multiple sequence recognition (*hsdS*) genes that recombine, resulting in distinct DNA methylation patterns and patterns of gene expression. These phenotypic switches have been linked to virulence and have recently been discovered in multiple clonal complexes of *L. monocytogenes*. In the present study, we investigated the significant of RMS on *L. monocytogenes* virulence during the acute phase of experimental meningitis.

**Methods:** *L. monocytogenes* strains containing RMS systems were identified, and purified clones enriched for single *hsdS* alleles were isolated. *In vivo*, 11-day old Wistar rats were infected with an inoculum containing (a) one of 4 single RMS allele variants (A, B, C, D) treated with amoxicillin (AMX 50 mg/kg/dosis, q8h), (b) a mixture of all 4 variants with or without AMX treatment, or (c) different mixtures of 2 RMS allele variants. At selected time points after infection, clinical and inflammatory parameters, bacterial titers and brain damage were determined. Changes in the relative frequency of the occurring RMS alleles in the inoculum and in CSF or cerebellum of infected animals were analyzed by capillary electrophoresis.

**Results:** We have identified a phase variable RMS locus within *L. monocytogenes* CC4 and generated stocks that stably expressed each of the possible *hsdS* genes within that loci. Generation of these allele variants (A, B, C, D) allowed us to determine the methylation pattern associated with each *hsdS* through SMRT sequencing. *In vivo* infections with these single allele variants revealed differences in disease severity in that C induced the worst clinical outcome and more pronounced hippocampal apoptosis; D showed the most pronounced weight loss and the highest bacterial titer in the cerebellum. A caused the least severe disease.

**Conclusion:** We identified that *L. monocytogenes* expressing *hsdS* (A) causes less damage than when other *hsdS* genes are expressed. While expression of *hsdSC* and *D* worsened the outcome in *L. monocytogenes* meningitis. We also demonstrate a competitive advantage of variants C and B over variant A in this model. Phenotypical switching may therefore represent a mechanism of virulence regulation during the acute phase of CNS infections with *L. monocytogenes*.

## Introduction

*Listeria monocytogenes*, a gram-positive facultative intracellular bacterium is a ubiquitous pathogen able to cause infections of the central nervous system (CNS), bacteremia involving the spleen and the liver, and localized gastrointestinal symptoms (Disson and Lecuit, 2012). Listeriosis primarily affects immunocompromised adults, the elderly and pregnant women via a foodborne infection route with high mortality rates of up to 50% (Yildiz et al., 2007; de Noordhout et al., 2014; Koopmans et al., 2017). In around 30% of the cases, listeriosis develops into bacterial meningitis and meningoencephalitis (Charlier et al., 2017). After *Streptococcus pneumoniae* and *Neisseria meningitidis, Listeria monocytogenes is* the third most common causative pathogen in meningitis/meningoencephalitis in around 5% of all cases (Koopmans et al., 2013).

Infections of the CNS by *L. monocytogenes* cause neurological sequela like epilepsy, hydrocephalus, strokes, severe intellectual disability and motor impairment (Kasanmoentalib et al., 2010; de Noordhout et al., 2014). In up to 18% of the patients, sequelae are still present 3 months after infection (Pelegrin et al., 2014). In neonates with a CNS infection, 44% developed neurological sequelae (de Noordhout et al., 2014). Complications like hydrocephalus occur in 28% percent of the cases (Hsieh et al., 2009) and is a risk factor for higher mortality (Pelegrin et al., 2014). Children are especially affected by long term disabilities. After 5 years 44% of all cases of neonatal neurolisteriosis still showed sequelae (Bedford et al., 2001).

The overshooting immunoreaction of the central nervous system (CNS) is an important driver in the pathophysiology of bacterial meningitis/meningoencephalitis and leads to poor outcome (Agyeman et al., 2014). Adults show a severe meningeal inflammation with a monocytic and granulocytic infiltration. Ventriculitis is frequently encountered and, often the periventricular tissue is being invaded. Commonly, *L. monocytogenes* meningitis leads to micro-abscesses. Those occur in proximity of the ventricles or perivascular spaces. Further the infection with *L. monocytogenes* leads to subendothelial inflammation of the meningeal arteries. Small vessel in the parenchyma show thrombosis and parenchymal bleedings (Engelen-Lee et al., 2018). The hippocampal formation of patients dying in association with bacterial meningitis show neuronal apoptosis of the granular layer in the dentate gyrus (Nau et al., 1999). This is also true in experimental models (Leib et al., 1996; Bifrare et al., 2003; Grandgirard et al., 2007b). The hippocampus is important for spatial and verbal learning and memory and damage within it leads to learning and memory impairment consecutive to bacterial meningitis (Nau et al., 1999; Wellmer et al., 2000; Loeffler et al., 2001; Leib et al., 2003).

The current therapy for listeria meningitis consists of amoxicillin, ampicillin or penicillin G. A duration of 21 days or longer is recommended, however there is no empiric data on an optimal treatment duration (Tunkel et al., 2004; van de Beek et al., 2016; Pagliano et al., 2017).

Phase-variation (PV), a rapid and reversible process, allows bacteria to diversify within populations and quickly adapt to changing environmental conditions. This is a mechanism with the potential to explain the ability of certain species, lineages or clones of pathogenic bacteria to be more successful in causing disease (Srikhanta et al., 2005; De Ste Croix et al., 2017, 2020a). PV DNA methylation systems have been proposed as one mechanism which could allow global gene expression changes through alternate methylation of the genome (Srikhanta et al., 2005; Manso et al., 2014). PV type I restriction modification systems (RMS) have been implicated in species such as *S. pneumoniae* (Manso et al., 2014; Li et al., 2016; Kwun et al., 2018; De Ste Croix et al., 2019, 2020b), *Streptococcus suis* (Atack et al., 2018), *Mycoplasma pulmonis* (Sitaraman et al., 2002) and *L. monocytogenes* (Fagerlund et al., 2016) as potential epigenetic regulators of bacterial virulence. In *L. monocytogenes*, as in many other bacterial species, RMS tend to be specific to the accessory genome of single phylogenetic units such as sequence type or clonal complex (Chen et al., 2017; De Ste Croix et al., 2017; Lee et al., 2019; Zamudio et al., 2020). Previously a PV type I RMS was described in *L. monocytogenes* lineage II sequence type 8 (ST8) isolates of food and human origin (Fagerlund et al., 2016). We identified a related phase variable type I RMS in *L. monocytogenes* lineage I ST4.

The aim of this work was to isolate strains stably expressing each of the PV *hsdS* genes of this type I RMS and characterize the associated methylome changes and their potential effects on disease associated phenotypes in an established infant rat model of experimental meningitis/neurolisteriosis.

## Materials and Methods

### Bacterial Strains, Growth Conditions, and Growth Curves

*L. monocytogenes* isolates of sequence type 4 (ST4) were clinical isolates from the Institute of Infectious Diseases in Bern and blood culture isolates collected by the National Reference Laboratory for Enteropathogenic Bacteria and Listeria (NENT) in Zürich (Table 1). Bacterial strains were grown at 37°C on brain heat infusion (BHI) agar plates or in BHI broth (Thermo Fisher Scientific, UK). Wildtype (wt) clones of *L. monocytogenes* N12-320 stably expressing a single RMS *hsdS* allele were generated. These clones were generated through the passaging of single colony isolates on BHI agar at 37°C, followed by PCR analysis of the *hsdS* locus using primers WRT3 and WRT4 as described below. For each *hsdS* allele 3 clones were independently generated and named A1-3, B1-3, C1-3, and D1-3. Each of these clones is a wt isolate capable of recombination within the RM system locus, however it is significantly enriched (>90%) for a single *hsdS* allele. Stable *hsdS* expressing clones were also generated in the CC4 strains 2250248 and N12-0794.

**Table 1:** *L. monocytogenes* strains used in this study.

**Strain**	**Origin**	**Source**	**Type**	**ST**	**Accession**	
2250248	Bern	CSF	4b	4	JACBGT000000000	This work
N12-0320	Zurich	Blood	4b	4	NZ_QYGX00000000.1	Althaus et al., 2014
N12-0794	Zurich	Blood	4b	4	NZ_QYGH00000000.1	Althaus et al., 2014
N13-0772	Zurich	Blood	4b	4	NZ_QYEK00000000.1	Althaus et al., 2014

Clones enriched for a single *hsdS* allele, from strain N12-0320, were used to confirm that all strains showed a similar growth rate *in vitro*. Strains were plated on BHI agar (Oxoid, UK) and incubated overnight at 37°C. For each strain a sweep of colonies was resuspended in BHI broth to an OD600 of ~0.4. An additional 25% mix containing A1, B1, C1, and D1 was generated by pooling strains in equal proportions by OD. Each sweep was then diluted 1:100 in fresh BHI broth. 200 ul per strain per replicate was aliquoted into a sterile, flat-bottomed, 96 well-plate (Thermo Fisher Scientific, UK) and sealed with a Breathe-Easy membrane (SigmaAldrich, UK). Growth was measured for 24 h in an Eon Biotek plate reader (Biotek, UK) at 37°C. Growth curve data was plotted using ggplot2 in R/4.0.2. Data from independent strains (e.g. A1, A2, A3) were used to generate error bars.

### Molecular Analysis

#### SMRT Sequencing

We determined the complete genome of *L. monocytogenes* N12-0794 *hsdSA* and N12-0794 *hsdSC* using single-molecule, real-time (SMRT) sequencing. In short DNA was prepared from exponential phase (OD0.4) cultures using the Zymo Clean & Concentrator DNA kit (Cambio, UK) and the manufacturers protocol. Sequencing libraries were prepared according to the manufacturer's instructions and sequenced on the PacBio Sequel II instrument. Genomes were assembled *de novo* and reads were analyzed for methylation patterns using SMRT analysis software (available from Pacific Biosciences). Analysis was conducted on CLIMB (Cloud Infrastructure for Microbial Bioinformatics) (Connor et al., 2016).

#### GeneScan of *hsdS* Alleles in Recovered Samples

Bacterial colonies obtained from intra-cisternal infections of Wistar Rat pups were analyzed for *hsdS* expression and compared to the initial inoculating dose. To ensure only live bacteria were analyzed all samples were plated on BHI agar and incubated at 37°C overnight. All bacterial growth was collected and used for PCR amplification of the phase variable region of interest with a FAM labeled primer (WRT4 5′-[6FAM]CCAGTAATCCGGTTTAAAGGC) and primer WRT3 (5′-CCAAGCGAATCTGTAGCCC) (Sigma, UK). Following successful PCR amplification of the phase variable region of interest, samples were digested using EcoRV (NEB, UK), giving a unique fragment size for each variant when in the active *hsdS* position (allele A 780, B 727, C 705, and D 756 bp). Fragment size analysis of FAM labeled, digested PCR products was done on an ABI Prism DNA sequencer using the LIZ1200 size standard (Thermo Fischer Scientific, UK). Size analysis of each labeled fragment was run in Peak Scanner v1.0 (Thermo Fisher, UK). For homogenized organs 20 ul was plated and for CSF samples (due to the small sample volume) 10 ul was plated. A minimum of 10 colonies were required for analysis to ensure samples were representative, samples with <10 colonies were not analyzed further. The initial inoculum for each experiment was also analyzed by plating and PCR of the recovered colonies. If two attempts to PCR amplify the locus of interest failed the sample was excluded from further study.

#### Phylogenetic Analysis

As we previously described, the core-genome phylogenetic tree for our Swiss isolates was build based on 1,596 core genes, which represent 93.8% of the well-defined cgMLST scheme (Zamudio et al., 2020). Gene markers for phase variable Modification Restriction systems (pv-MRS) were identified in our 160 Swiss isolates by using BLASTn with 100% coverage and 98% identity. The gene presence/absence matrix was mapped into the phylogenetic tree using the ggtree R package v1.15 (Yu et al., 2017).

### Bacteria Preparation for *in vivo* Infections

Clones enriched for a single *hsdS* allele, isolated from the strain N12-0320, were used in all *in vivo* experiments. The bacteria stored at −80°C on ceramic beads were stroked on Columbia sheep blood agar and incubated overnight at 30°C. Single colonies were picked up and grown at 30°C in prewarmed brain heart infusion broth (BHI) for 18 h, under static conditions. After that, bacteria were pelleted at 1,560 rcf (Heraeus Biofuge Fresco, Thermo Fischer Scientific, Waltham, Massachusetts, US) for 10 min at 4°C. The pellet was resuspended in sterile NaCl 0.85%. This procedure was repeated twice. The resuspended bacterial preparation was diluted in sterile NaCl 0.85% and adjusted to the desired concentration by measuring the optical density at 570 nm. Inoculum size accuracy was checked by quantitative culture on blood agar plates. For *in vivo* experiments, 10 μl of the suspension were injected intracisternally.

### Experimental Model of Neurolisteriosis

All animal studies were approved by the Animal Care and Experimentation Committee of the Canton of Bern, Switzerland (license no. BE 1/18). The model used in the present study was previously established in our group (Michelet et al., 1999; Remer et al., 2001). Per experiment 14 eleven-day old rat pups of mixed sex and one dam were purchased from Charles Rivers (Sulzfeld, Germany). The dams were provided with tap water and pellet diet *ad libitum*. Litters were kept in rooms at a controlled temperature of 22 ± 2°C. The average weight of the animals upon start of the experiments was 22.0 ± 2.7 g. Our experimental model with 11-day old rats, corresponds to term infants or very young children <1 year old in term of brain development (Semple et al., 2013). This model is therefore well-suited to investigate both the acute phase of the disease and later neurofunctional deficits.

Via a 30-gauge needle (Becton Dickinson Microlance™, Allschwil, Switzerland) 10 μl of the bacteria suspension was injected into the cisterna magna of infant rats. The animals were weighted, clinically scored according to this scheme (1 = coma, 2 = does not turn upright, 3 = turns upright in >5 s, 4 = turns upright in <5 s, 5 = normal) and CSF was gained by puncturing the cisterna magna at different times points (18, 24, and 42 h post infection, hpi). These time points correspond to (a) a time preceding the symptoms appearance, when we begin to monitor the animals (18 hpi), (b) initiation of antibiotic therapy, with a significant worsening of clinical symptoms (24 hpi), and (c) to the time of sacrifice, when we investigate the development of acute brain damage by histology. Depending on the experiment (see next paragraph), animals were treated with either Amoxicillin (AMX 50 mg/kg/dosis, q8h) or saline, starting at 24 hpi. Animals were checked regularly to evaluate their health status by determining their clinical score. When reaching a clinical score <2, animals were euthanized for ethical reasons. At 42 h after infection, surviving animals were sacrificed with an overdose of the anesthetic pentobarbital (Esconarkon, Streuli Pharma AG, Uznach, Switzerland 200 mg/kg body weight, i.p.,). Animals were perfused through the left ventricle with ice-cold phosphate buffered saline (PBS). Brains were removed for later histological processing. The cerebellar samples were frozen on dry ice and stored at −80°C for later homogenization.

### Experimental Design

#### Infections With Single RMS Allele Variants

Animals (*n* = 36) received an inoculum prepared from a clone enriched for a single *hsdS* allele as described above. Inoculum sizes were 2.03 ± 0.55 × 10^7^ cfu/ml for variant A (*n* = 3), 1.70 ± 0.60 × 10^7^ cfu/ml for B (*n* = 3), 2.19 ± 1.52 × 10^7^ cfu/ml for C (*n* = 3), and 2.46 ± 1.65 × 10^7^ cfu/ml for D (*n* = 3). The assignment of the animals for infection with one of the variants was randomized with 9 animals infected per variants. All animals were treated starting at 24 hpi with Amoxicillin (AMX 50 mg/kg/dosis, q8h). Two animals had to be sacrificed early, due to traumatic puncture injuries (B: *n* = 1; D: *n* = 1) and two animals died spontaneously from infection (A: *n* = 1 and C: *n* = 1). Thirty-two animals reached the endpoint of the experiment at 42 hpi (A: *n* = 8, B: *n* = 8, C: *n* = 8, D: *n* = 8).

#### Infections With a Mixture of the 4 RMS Allele Variants (A:B:C:D)

Animals (*n* = 28) received an inoculum prepared from a mix of 4 clones enriched for the different *hsdS* alleles. The inoculum size was 1.80 ± 0.35 × 10^7^ cfu/ml (*n* = 3). The animals were randomly assigned to a treatment with AMX (50 mg/kg/dosis, q8h) or saline starting at 24 hpi. The assignment to the treatment group was randomized (AMX *n* = 14, saline *n* = 14). Three animals spontaneously died in the control group and one animal of the AMX group had to be sacrificed early due to a traumatic puncture injury, so that a total of 24 animals reached the endpoint of the experiment at 42 hpi and were further included in the analysis data (AMX = 13, control = 11). Apart from clinical, histological and inflammatory parameters, the *hsdS* expression (A:B:C:D) within the bacterial colonies recovered from the CSF and cerebellum was compared to the original inoculum by analysis of PCR fragments (GeneScan).

#### Infections With a Mixture of 2 RMS Allele Variants (A:B, A:C, B:C)

Animals received an inoculum prepared from a mix of 2 clones enriched for *hsdS* alleles A and B (A:B, *n* = 36), alleles A and C (A:C, *n* = 24) or alleles B and C (B:C, *n* = 24). The inoculum size was 1.46 ± 0.32 × 10^7^ cfu/ml for A:B (*n* = 3), 1.03 ± 0.11 × 10^7^ cfu/ml for A:C (*n* = 4) and 1.16 ± 0.37 × 10^7^ cfu/ml for B:C (*n* = 4). No treatment was applied for these experiments. Twenty-five animals reached the endpoint of the experiment at 42 hpi for A:B, 16 for A:C and 15 for B:C. The *hsdS* expression of the different alleles (A, B, C, and D) within the bacterial colonies recovered from the CSF and cerebellum was compared to the original inoculum by analysis of PCR fragments (GeneScan).

### Quantitative cfu Determination

CSF samples were serially diluted in sterile NaCl 0.85% and plated on CSBA plates. To prepare cerebellar homogenates, 1 ml of NaCl 0.85% was added per gram of tissue and mechanically processed in a glass homogenizer. The homogenate was serially diluted and plated on CSBA plates. All plates were cultured at 37°C overnight. Results were expressed as cfu/ml for CSF samples, respectively, cfu/g for homogenized tissue.

### Analysis of Cytokine Expression in Cerebellar Homogenates

Cytokines known to be upregulated during *L. monocytogenes* meningitis (IL-1β, IL-6, IL-10, IL-18, TNF-α, and VEGF) (Koopmans et al., 2014, 2018) were assessed using magnetic multiplex assay (Rat Magnetic Luminex® Assay, Rat Premixed Multi-Analyte Kit, R&D Systems, Bio-Techne) on a Bio-Plex 200 station (Bio-Rad Laboratories) as described previously (Perny et al., 2016; Muri et al., 2018). Cerebellum homogenates were centrifuged (16,000 × g, 10 min, 4°C) and protein concentration determined using Pierce™ BCA Protein Assay kit (ThermoFischer Scientific). 100–150 μg proteins were diluted to a final volume of 50 μl. For each sample, a minimum of 50 beads was measured. If the concentration of the sample was below the detection limit, a value corresponding to the lower limit of detection provided by the manufacturer was used. The detection limits for undiluted samples were 2.93 pg/ml for IL-1β, 23.2 pg/ml for IL-6, 8.95 pg/ml for IL-10, 3.32 pg/ml for IL-18,11.5 pg/ml for TNF-α, and 15.6 pg/ml for VEGF.

### Histopathology

Brain harvested at sacrifice were fixed for 4 h in 4% PFA and cryo-preserved in a 18% sucrose in PBS at 4°C for 24 h. After freezing the brains in methyl-butane at −80°, forty-five μm slices were cut and every 15th cut was mounted on a gelatin coated glass slide. The slides were stained with cresyl violet (Merck, Zug, Switzerland) for Nissl bodies and mounted with DPX mounting medium for Histology (Sigma-Aldrich, Buchs, Switzerland). The sections were analyzed for hippocampal apoptosis as described previously (Grandgirard et al., 2007a). Hydrocephalus was assessed by determining the volume of the lateral and third ventricles normalized to the respective total cortical volume. All volumes were determined using the Cavalieri principle (Grandgirard et al., 2007a) using Image J for the analysis (V. 1.45, National Institutes of Health, Bethesda, Maryland, US). In our hand, a 15th cutting frequency allows enough resolution to determine volumes using histology, with negligible variation compared to water displacement method. Histologic assessment was performed and evaluated by investigators blinded to treatment modalities of the individual animals. Digital pictures of the dentate gyrus were taken using an AxioImager M1 microscope equipped with an AxioCam MRc CDD camera (Carl Zeiss Microscopy, Göttingen, Germany) at a magnification of 40x. Entire histological slices were scanned using a Path Enabler IV scanner (Meyer Instruments Inc., Houston, TX, US) at a resolution of 3,600 dpi.

### Statistical Analyses

Statistical analyses were performed using GraphPad Prism (Prism 8; GraphPad Software Inc., San Diego, USA). Results are presented as mean values ± standard deviation if not stated otherwise. Survival was calculated using a log rank (Mantel-Cox) test. To compare differences between two groups, an unpaired Student *t*-test or a non-parametric Mann-Whitney test were used. When comparing multiple groups, we performed either one-way ANOVA with Tukey multiple comparison or a Kruskal-Wallis with Dunn's multiple comparison test depending on the normal distribution of the groups. For data available for different time points we performed a mixed-effects model of a 2-way ANOVA with repeated measures (because of missing values for animals that died spontaneously) and Tukey multiple comparison. The active *hsdS* allele composition of each sample was compared to the initial inoculum using a Kruskal-Wallis test. Normal distribution was tested using the D'Agostino & Pearson test. A *p* < 0.05 was considered statistically significant with *p* < 0.05 (^*^), *p* < 0.01 (^**^), *p* < 0.001 (^***^), and *p* < 0.0001 (^****^).

## Results

### Genetic, Genomic, and Phenotypic Analysis of the Phase Variable Type I RMS

**Figure 1:**
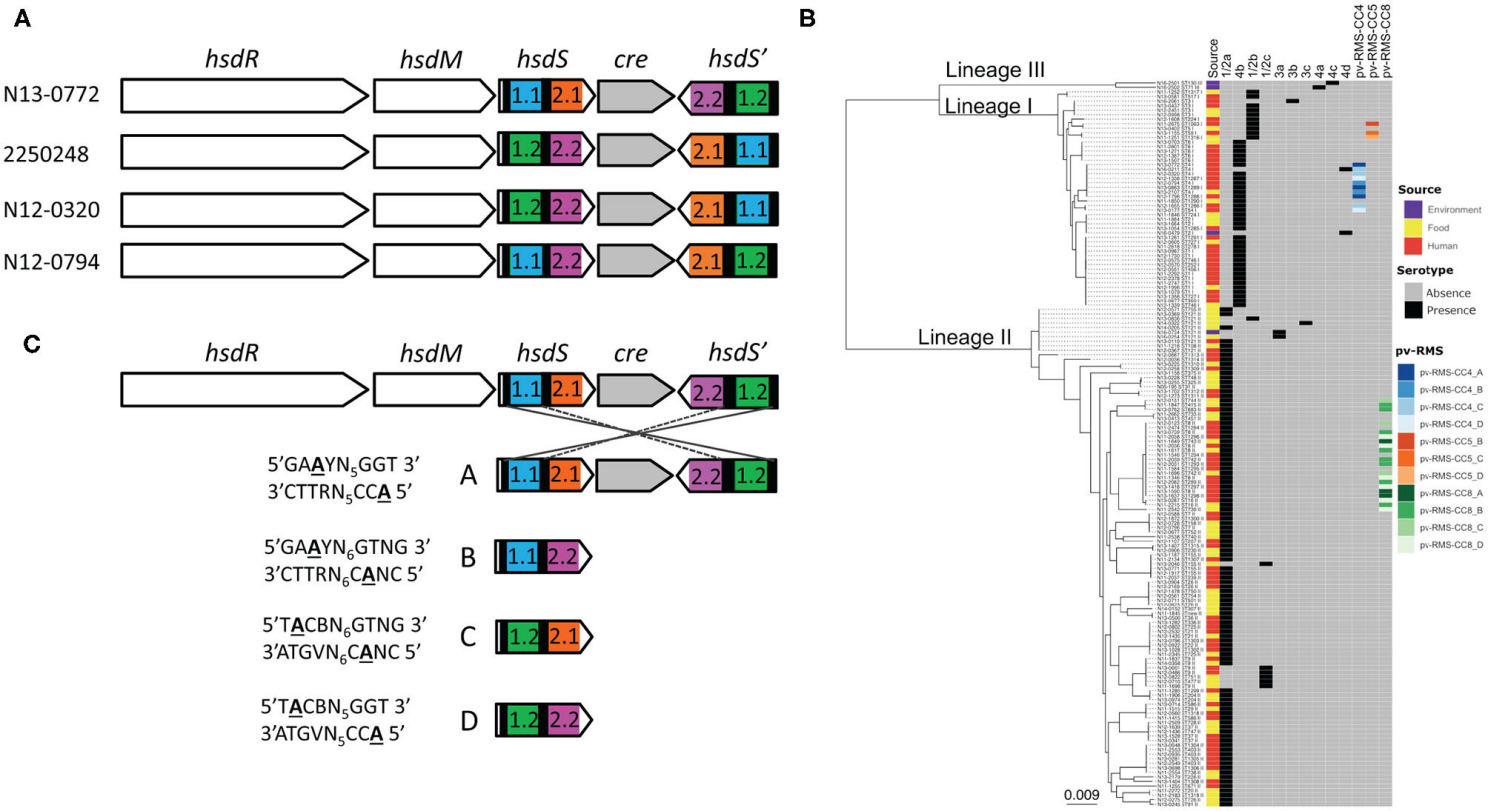
The phase variable Lmo0320I RMS in *L. monocytogenes* CC4. The locus of the Lmo0772I RMS is composed of an operon containing the co-transcribed *hsdR* (restriction subunit), *hsdM* (methylation subunit) and *hsdS* (specificity subunit) genes, a site-specific recombinase (gray), and a further inactive *hsdS* gene without a proper start codon and no promoter. The alignment of the locus for the phase variable RMS from four Swiss ST4 CC4 isolates indicates how recombination on the inverted repeats within the *hsdS* genes (black bars) is able to shuffle the target recognition domains TRD1.1 (blue), 1.2 (green), 2.1 (orange), and 2.2 (pink) **(A)**. To investigate the phylogenetic context all *L. monocytogenes* phase variable RMSs were mapped on a core-genome phylogenetic tree of 160 Swiss *L. monocytogenes* isolates (Zamudio et al., 2020). The color of the squares in the heat map are linked to the source (human in red, food in yellow and environment in purple), serotype (presence in black and absence in gray) and phase variable RMS (pv-RMS-CC4 in blue, pv-RMS-CC5 in orange and pv-RMS-CC8 in green. The active variant (A, B, C, or D) of each pv-RMS is indicated by a different shading. **(B)** Rearrangements within the locus generate the four possible hsdS alleles, A–D by creating different combinations of TRDs 1.1 (blue), 1.2 (green), 2.1 (orange) and 2.2 (pink). Each TRD combination results in an alternative methylation pattern of the genome. These methylation patterns were determined by SMRT sequencing and are shown to the left of each hsdS allele **(C)**.

For the genetic and genomic analysis of the phase variable type I RMS Lmo0320I in *L. monocytogenes* lineage I ST4 (sequence type 4, clonal complex 4 CC4) isolate N13-0320, we aligned the RMS system loci of four ST4 genomes (Figure 1A). The 8,446 bp phase variable RMS locus was found to be inserted into a hairpin in the intergenic between lmo020 and lmo0521 in the reference strain EGD-e (between GeneID 985284 and 985300) creating a deletion of 32 bp. The structure of the locus was maintained between all CC4 isolates with an *hsdR* gene coding for the restriction subunit, an *hsdM* gene encoding for the methyltransferase, and one active and one inactive *hsdS* gene encoding for the specificity subunit, interspaced by the gene for a site-specific recombinase with a 99% nucleotide identity spanning the whole locus. As already shown for other phase variable type I RMS loci (Manso et al., 2014; Fagerlund et al., 2016), shuffling of the single target recognition domains (TRD) of the *hsdS* genes is readily evident from the aligned genome sequences (Figure 1A). Testing our collection of 160 Swiss *L. monocytogenes* isolates of food and clinical origin (Althaus et al., 2014) shows presence of the phase variable RMS Lmo0320I in all eight CC4 isolates and the related isolates of ST54 and ST1286 (Figure 1B). Further interrogation of our set of genomes with the sequences of the Lmo0320I *hsdR* genes detected orthologous systems in CC5 and CC8 with sequence identity, respectively, of 3063/3063 (100%) (Lineage I CC5 isolate N13-0402, accession RDSW00000000) and 3015/3063 (98%) (Lineage II CC8 isolate N11-2036 accession QYHX00000000) (Figure 1B). The *hsdM* genes shared a similar level of sequence identity. Three of the Lmo0320I target recognition domains were present in CC5, namely TRD 1.2, 2.1, and 2.2 (nucleotide identity 99–100%). In the more distantly related lineage II CC8 RMS (Fagerlund et al., 2016), only one of the four TRDs was partially conserved (TRD1.1, identity 642/695 92%).

Analysis of *hsdS* allele prevalence in single colonies of N12-0320, N12-0794, and 2250248 showed that it was possible to isolate colonies, and stocks derived thereof, which expressed prevalently different *hsdS* alleles. Allele quantification showed that over 95% of cells in overnight grown colonies (16 generations) had the same active *hsdS* allele (data not shown), indicating that in this condition recombination in the locus was a rare event. These enriched stocks were used for SMRT sequencing. Methylome analysis identified four different methylation targets for the four different *hsdS* alleles (Figure 1C). In strain N12-0320, there are a total of 620 *hsdSA*, 531 *hsdSB*, 597 *hsdSC*, and 484 *hsdSD* methylation sites, representing a huge range of genes potentially regulated by a single type I RMS.

The growth rates of the N12-030 strains expressing different alleles, used for all *in vivo* work in this study, were analyzed *in vitro*. All strains showed a similar growth rate (Supplementary Figure 1A).

### Infections With Single RMS Allele Variants

All results presented in this paragraph are also summarized in Supplementary Table 1.

#### Clinical Parameters

**Figure 2:**
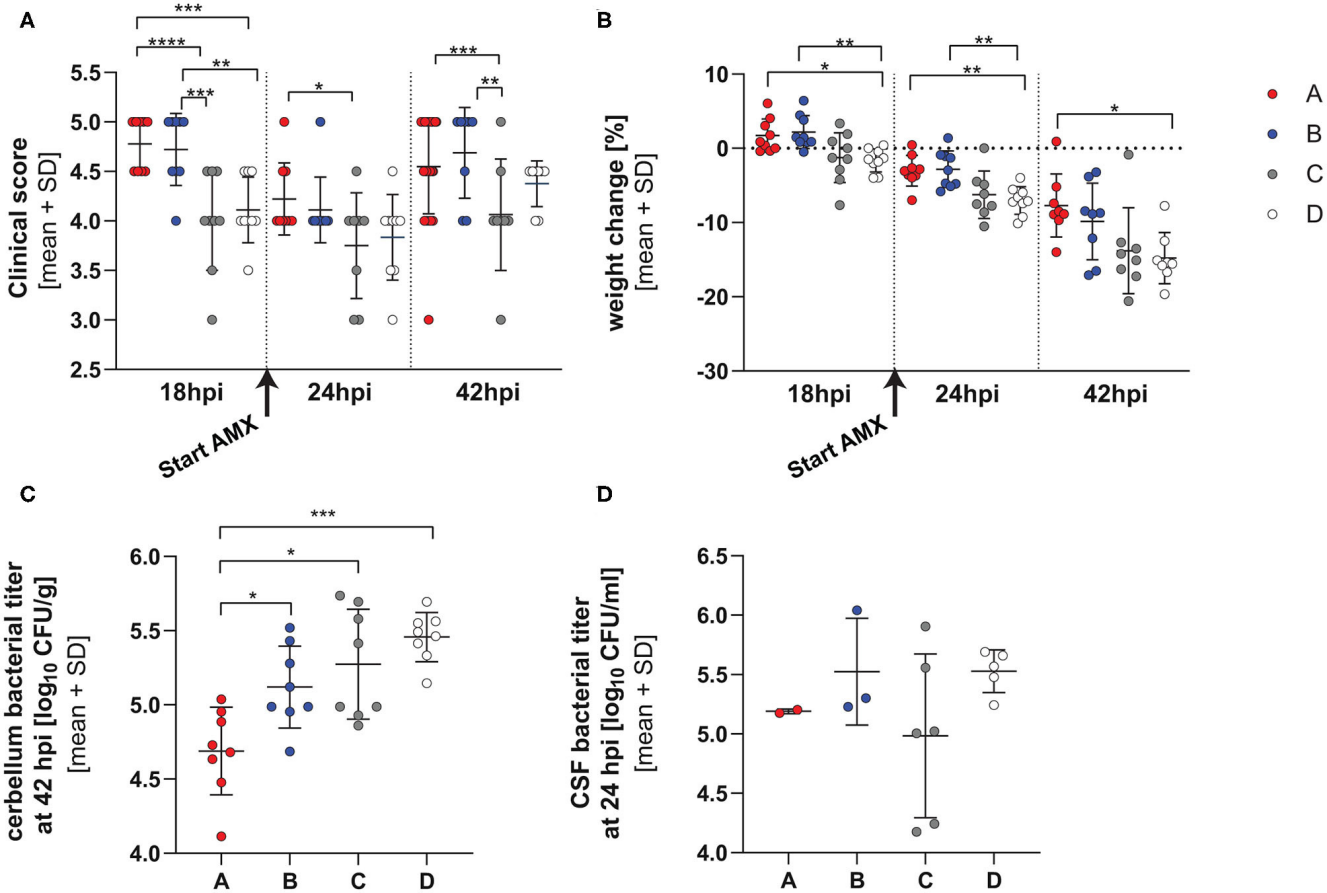
Clinical parameters in infection with single RMS allele variant. Clinical score **(A)**, weight change **(B)** and bacterial titers in cerebellum at 42 hpi **(C)** and CSF at 24 hpi **(D)** in animals infected with the 4 different *hsdS* allele variants (A: *n* = 8, B: *n* = 8, C: *n* = 8, D: *n* = 8). The vertical dotted lines represent separation between the different time points and the initiation of the amoxicillin treatment is indicated by an arrow under the x-axis. The variants show altered virulence measured by the clinical score, weight change and cerebellum titer. The detailed statistical analyses are provided in Supplementary Tables 1, 2. *p < 0.05; **p < 0.01; ***p < 0.001; ****p < 0.0001.

All animals developed meningoencephalitis, as demonstrated by a worsening of clinical score and weight loss over time and growth of bacteria in the cerebellum (Figures 2A–C).

Animals infected with the different variants didn't significantly differ in mortality (Supplementary Figure 1B). Significant differences in clinical scores were observed between animals infected with the different strains at 18, 24 h, and 42 hpi, with those infected with variant A having consistently better clinical outcome scores at all time points and C significantly worse (Figure 2A and Supplementary Table 2 for detailed statistical analysis). After the application of AMX, there was an improvement of clinical signs for all variants.

Animals infected with all variants showed pronounced weight loss over time. In contrast to its effect on clinical score, AMX treatment didn't attenuate weight loss over time. By comparing the different strains, infection with variant A and B caused the smallest weight change at all time points whereas variant D caused the most pronounced weight loss over the duration of the experiment (Figure 2B and Supplementary Table 3 for detailed statistical analysis).

Cerebellum homogenates at 42 hpi showed significantly higher bacterial titers for animals infected with variants B, C, and D compared to variant A, and variant D showed the highest titer overall (*p* value for the comparison allele A vs. B, pAvsB = 0.0263, pAvsC = 0.0018, pAvsD < 0.0001, Tukey multiple comparison test) (Figure 2C).

No differences (*p* = 0.2886, one-way ANOVA) in bacterial titers could be observed in the CSF harvested at 24 hpi, before initiation of antibiotic therapy. However, CSF could be harvested only in a subset of the animals (Figure 2D) (variant A 1.555 ± 0.0707 × 105 cfu/ml *n* = 2, B 4.896 ± 5.287 × 105 cfu/ml *n* = 3, C 2.335 ± 3.062 × 105 cfu/ml *n* = 6, D 3.582 ± 1.265 × 105 cfu/ml *n* = 5).

#### Inflammatory Parameters

**Figure 3:**
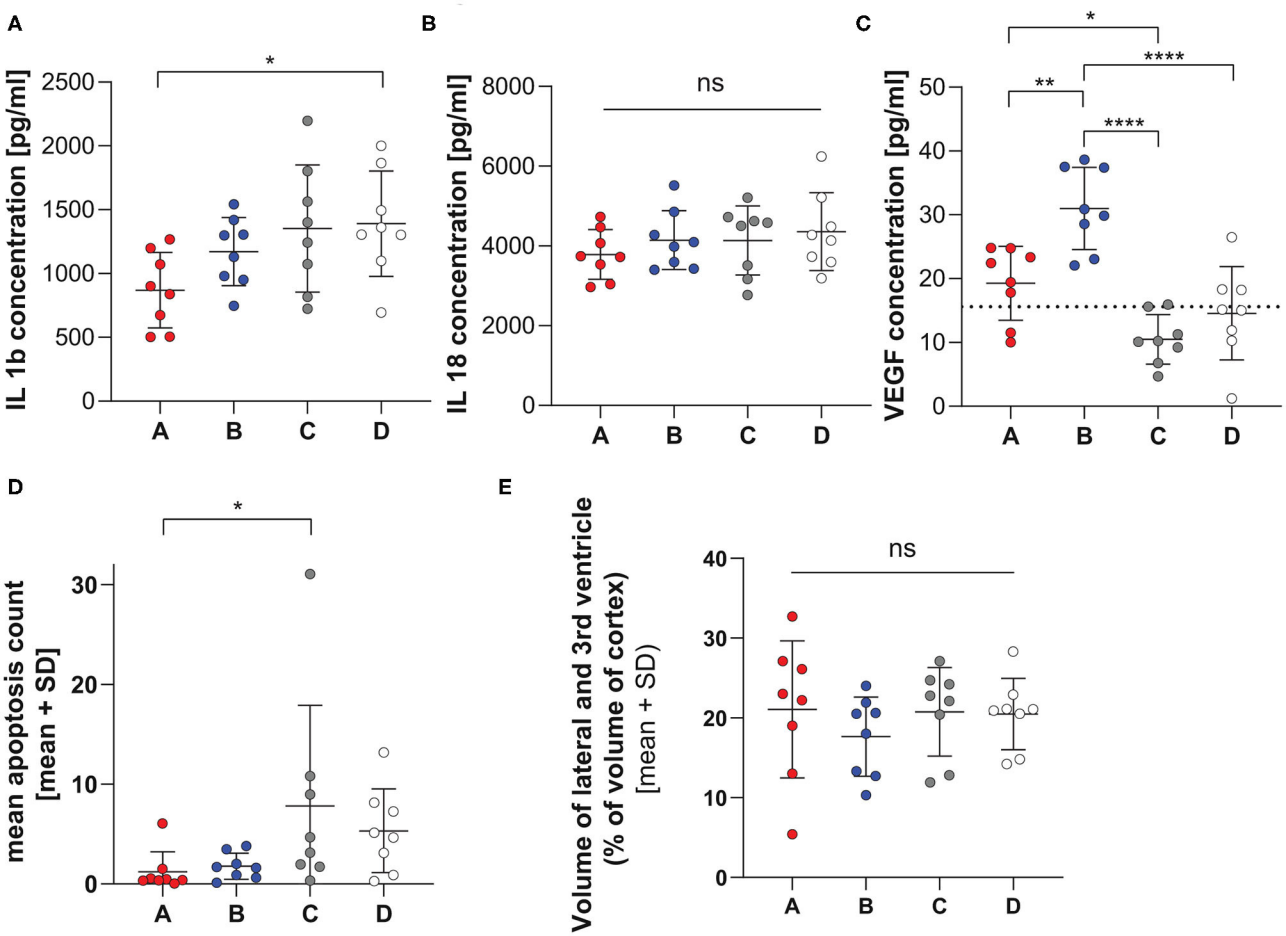
Inflammatory and histological parameters in infection with single RMS allele variant. Cytokine levels of IL-1β **(A)**, IL-18 **(B)**, and VEGF **(C)** measured in cerebellum homogenate at 42 hpi in infant rats infected with the 4 different *hsdS* allele variants (A: *n* = 8, B: *n* = 8, C: *n* = 8, D: *n* = 8). The horizontal dotted line represents the lower limit of detection, data points under the line are extrapolated measurements. The variants show different concentrations for IL-1β and VEGF. Variant D leads to higher IL-1β level, whereas variant B shows elevated VEGF levels. No significance was found for IL-18 levels. Significant difference in the level of apoptosis **(D)** was only found for the comparison between infection with variants A and C, while hydrocephalus **(E)** was not different between infection with the 4 variants. *p < 0.05; **p < 0.01; ****p < 0.0001.

The levels of different cytokines were investigated in cerebellar homogenates at 42 hpi (Figures 3A–C). A significant lower level of IL-1β concentration was observed in animals infected with variant A compared to those infected with D (*p* = 0.0488, Tukey's multiple comparison test). No further difference between the groups could be reported. No difference in IL-18 concentration was observed in animals treated with the different variants. Animals infected with variant C and D resulted in lower VEGF expression than A or B (pAvsC = 0.0319, pBvsC < 0.0001; pBvsD < 0.0001, Tukey's multiple comparison test). Further infection with variant B resulted in higher VEGF level than variant A (pAvsB = 0.0028). The level of matrix-metalloproteinase 9 (MMP9) was not different in animals infected with the different strains (Supplementary Figure 2).

#### Histology

Animals infected with variant A show less apoptotic cells in the hippocampus compared to animals infected with variant C. A trend for less severe apoptotic damage was also found for variant A compared to variant D (pAvsC = 0.0500, pAvsD = 0.0792, Dunn's multiple comparison test, Figure 3D and Supplementary Figure 3).

All animals showed enlarged ventricles. However, we did not find any significant difference in hydrocephalus for animals infected with the different variants (*p* = 0.6670, one- way ANOVA, Figure 3E and Supplementary Figure 3).

### Infections With a Mixture of the 4 RMS Allele Variants (A:B:C:D)

All results presented in this paragraph are also summarized in Supplementary Table 1.

#### Clinical Parameters

**Figure 4:**
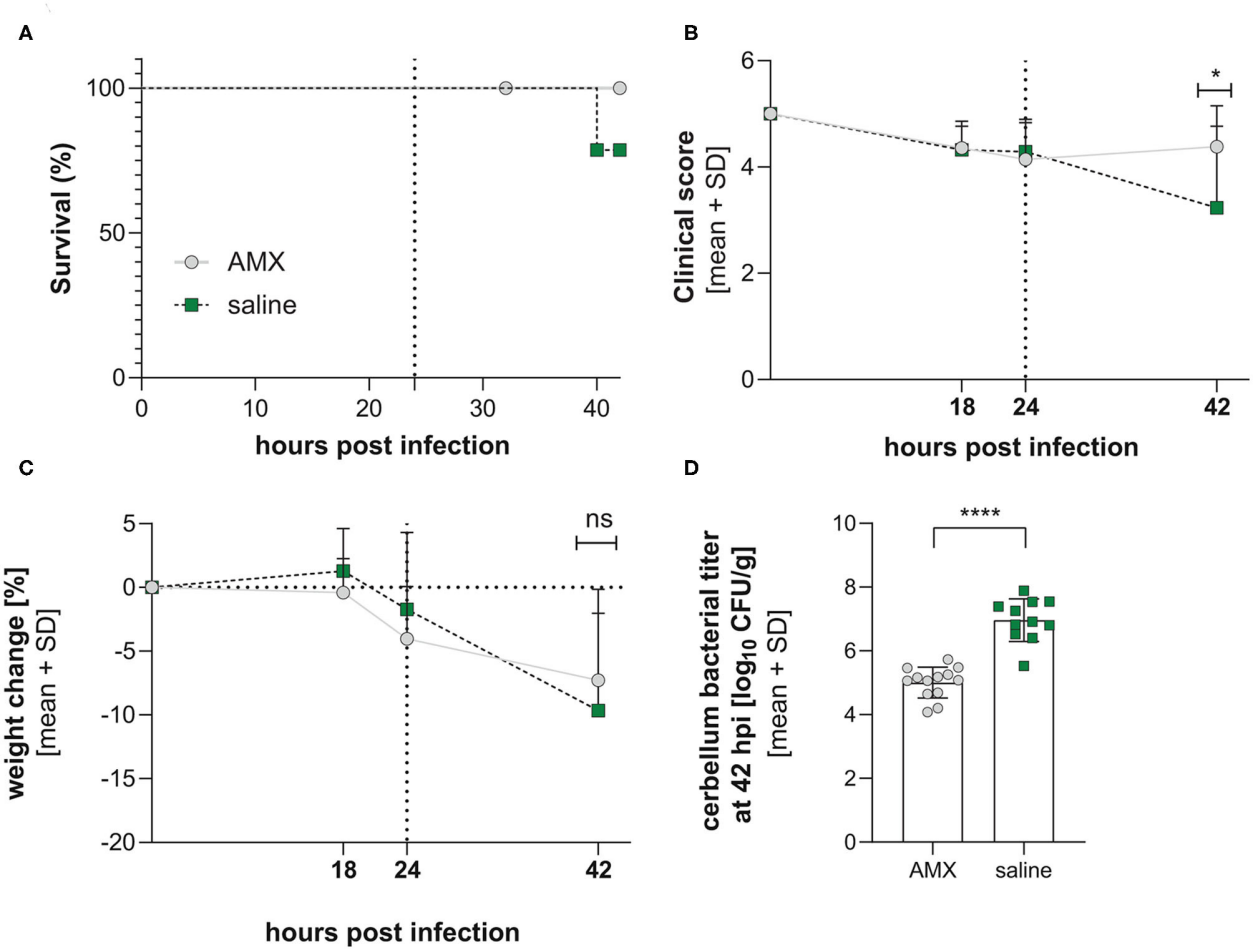
Clinical parameters in infection with a mixture of the 4 RMS allele variants. Comparison between AMX-treated and saline-treated animals didn't reveal changes in survival **(A)** (AMX = 14, control = 14). In contrast, clinical score **(B)**, weight change **(C)**, and bacterial titer in the cerebellum **(D)** were significantly improved by AMX treatment with amoxicillin improves outcome (AMX = 13, control = 11). Statistical significance was reached for clinical score and cerebellum titers. The vertical dotted line represents the initiation of the amoxicillin treatment. *p < 0.05; ****p < 0.0001; ns: not significant.

Although 3 animals died prematurely in the saline-treated group, the treatment did not alter survival over 42 hpi (*p* = 0.0826, Mantel-Cox, Figure 4A). The infection induced an initial worsening of the clinical score up to 24 h post infection (hpi) and of weight loss up to 42 hpi. The treatment with AMX administered after 24 hpi significantly improved clinical score compared to saline at 42 hpi (*p* = 0.0317, unpaired *t*-test), but did not significantly attenuate weight loss (Figures 4B,C).

Bacterial titers investigated in cerebellum homogenates at 42 hpi showed a significantly lower bacterial titers for animals treated with AMX (*p* < 0.0001, unpaired *t*-test, Figure 4D).

**Figure 5:**
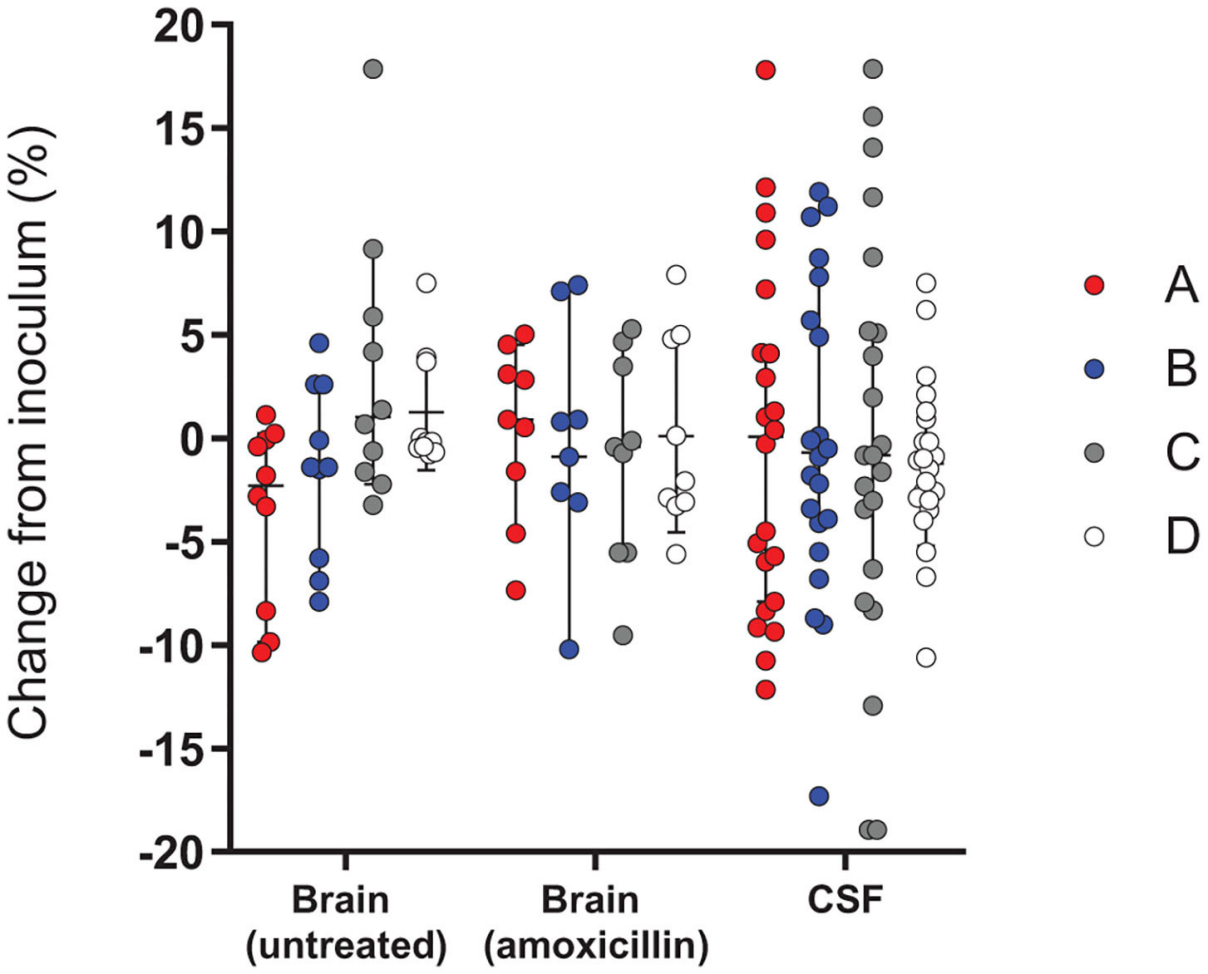
Change in frequency of the expressed RMS allele between the inoculum and different *in vivo* samples in infection with a mixture of the 4 RMS allele variants. The relative proportions of *hsdS* variants A (red), B (blue), C (gray) and D (white) between the inoculum and the bacteria harvested in the different i*n vivo* samples, including the brains of untreated animals (*n* = 10) or AMX-treated animals (*n* = 9) and CSF samples (*n* = 22), were not significantly different between the alleles. A Kruskal-Wallis test found no significant differences between variant proportions in the inoculums and *in vivo* samples.

Analysis of the colonies recovered from the CSF and brains of rat pups infected with the inoculum containing all four *hsdS* clones in relatively equal proportions allowed us to determine if any had a phenotypic advantage within a host meningitis model. To account for the variations in inoculum composition between the independent experiments, we calculated the change in proportion for each allele between the inoculum and the different *in vivo* samples, as determined by PCR. All CSF samples were collected prior to antibiotic treatment with amoxicillin, and brain samples were analyzed by separating the treatment groups. In this experimental paradigm, we did not observe any difference between the alleles, either in homogenized brain tissue or in the CSF (Figure 5).

#### Inflammatory Parameters

Except for VEGF, cytokines levels in cerebellum homogenates were significantly more elevated in saline treated animals compared to AMX treatment (IL-1β, *p* < 0.0001; IL-6, *p* = 0.0065; IL-10, *p* = 0.0095; TNF-α, *p* = 0.0033) (Supplementary Figure 4).

#### Histology

**Figure 6:**
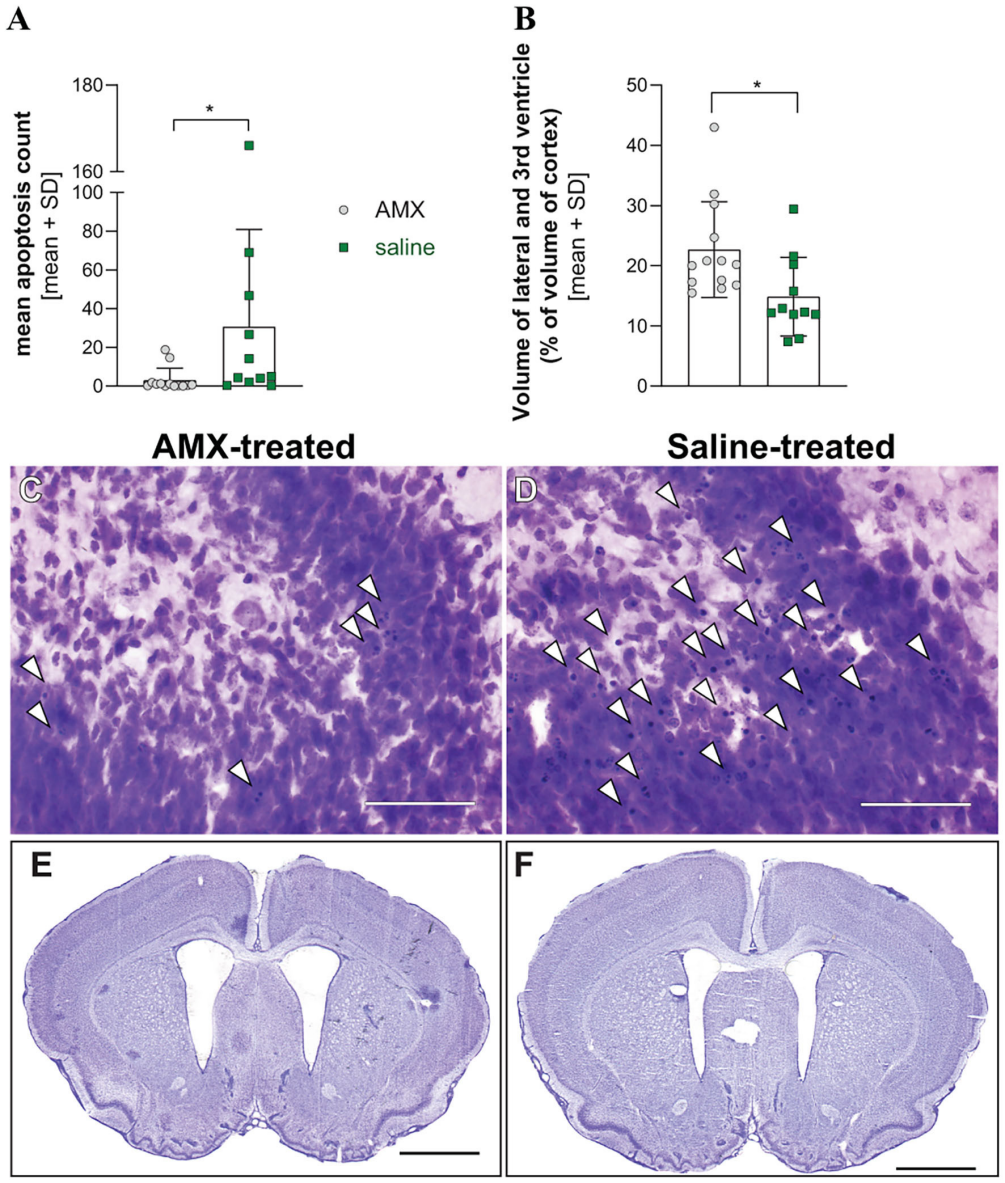
Histological parameters in infection with a mixture of the 4 RMS allele variants. Treatment of infected animals with AMX (*n* = 13) attenuated hippocampal apoptosis in comparison to saline-treated animals (*n* = 11) **(A)** but was associated with enlarged ventricle size **(B)**. Representative pictures of cresy violet-stained sections of the apex of the dentate gyrus (**C,D**, bar size 50 μm), with examples of apoptotic cells indicated with white arrowheads, or from entire sections with enlarged lateral ventricles (**E,F**, bar size 2 mm). *p < 0.05.

Animals who received AMX treatment suffered less hippocampal damage compared to animals who were treated with saline (*p* = 0.0107, Mann Whitney test, Figures 6A,C,D). Unexpectedly, animals treated with AMX had significantly more severe hydrocephalus than animals treated with saline (*p* = 0.0163, unpaired *t*-test) (Figures 6B,E,F).

### Infections With a Mixture of 2 RMS Allele Variants (A:B, A:C, and B:C)

The competition assay was repeated, but by reducing the mix to only two variants, A:B, A:C, or B:C. The animals were left untreated. All animals developed meningoencephalitis, proven by growth of bacteria in the cerebellum at 42 hpi and a worsening of clinical symptoms (not shown).

**Figure 7:**
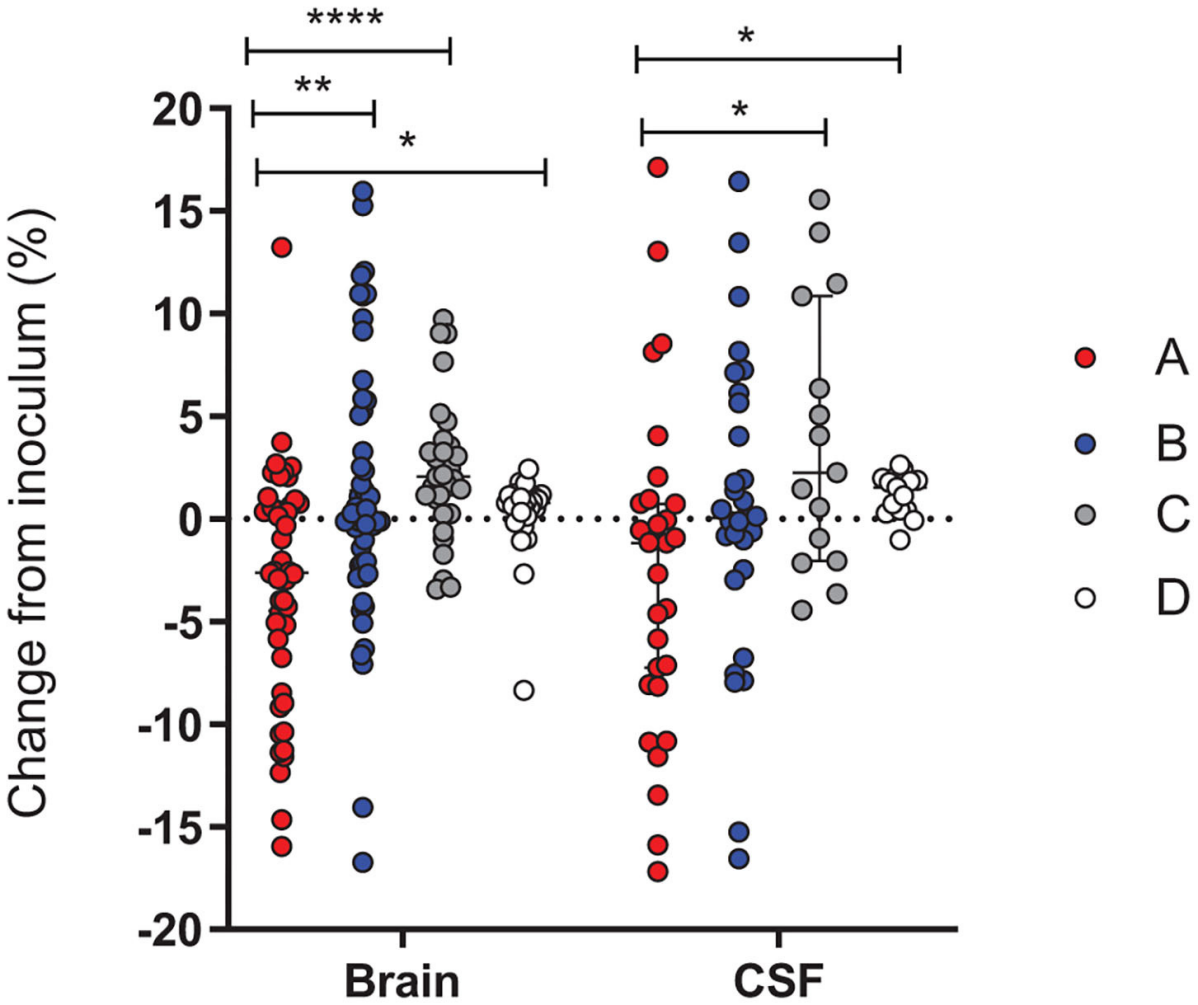
Change in the proportion of PV RMS alleles between the 2 variants inoculums and *in vivo* samples. Results were pooled from seven independent experiments and each variant is independently calculated as a percentage change from the initial inoculum for that experiment. The relative proportions of *hsdS* variants A (red), B (blue), C (gray) and D (white) between the inoculum and the bacteria harvested in *In vivo* CSF and cerebellum samples. A Kruskal-Wallis test showed that the proportion of the A variant is repeatedly reduced in comparison to the starting inoculum, while the C (CSF and brain) and B (brain) variants increase. *p* < 0.05 (*), *p* < 0.01 (**), and *p* < 0.0001 (****).

Similar to the experiment with the mix of 4 variants, we determined the changes in the proportion of different alleles between the inoculum and the different *in vivo* samples. Analysis of the recovered colonies, when compared to the inoculum, revealed that in both the brain tissue and in the CSF, variant A was significantly less represented when compared to the inoculum (Figure 7). This decrease was not observed with the other variants. Due to the nature of analysis a decrease in one variant is accompanied by an increase in an alternative variant. In both the brain and CSF, the observed reduction in variant A is accompanied by an increase in variant C (*p* < 0.0001 and *p* = 0.0166, respectively). In addition, the grouped comparison reveals that within the cerebellum the increase in the variant C is also at the expense of the variant B (*p* = 0.0226) while B appears to outcompete A (*p* = 0.008) (Figure 7). While no specific assay was conducted with the variant D, inoculums contained up to 10% D due to the wildtype nature of the strains. The results obtained are in line with those of the single variant infection experiments, showing significantly lower cerebellar titer for variant A in comparison to B and C (Figure 2C).

## Discussion

In the present study, we identified a phase-variable RMS system in a collection of clinical isolates of *Listeria monocytogenes* strains. While not as ubiquitous as some PV RMS (Manso et al., 2014), there appears to be a clear, clonal complex specific, distribution of PV RMS in *L. monocytogenes*. Analysis of the 160 genomes recently published by Zamudio et al. (2020) showed that all CC4 strains contain different orientations of the same loci (Figure 1B). Isolation of clones expressing a single *hsdS* variant in strains N12-0320, N12-0794, and 225078 allowed us to confirm that changes in *hsdS* expression are on-going and relatively stable at 37°C. Long read sequencing of N12-0320 allowed us to identify different methylation patterns associated with each *hsdS* (Figure 1C).

We tested clones enriched in one of the four allelic variants derived from a CC4 serotype 4b clinical isolate that displays this phase variable RMS in a neonatal rat model. Such phase variation mechanisms have been described in *S. pneumoniae* and are shown to be important in virulence in pneumococcal invasive diseases (Manso et al., 2014). While in *Hemophilus influenzae* a PV type III RMS has been linked to virulence phenotypes such as antibiotic resistance, biofilm formation and evasion of the immune system (Atack et al., 2015). Here we show that phase variation in type I RMS in *L. monocytogenes* leads to an altered outcome in our animal model of listeria meningitis.

When comparing animals infected with the single RMS allele variants, infection with variant A show the mildest clinical disease phenotype on different parameters, including clinical score, weight loss, bacterial titer in the cerebellum, cytokines in cerebellum homogenate and hippocampal apoptosis. Mortality was however not significantly different between all groups up to 42 h after infection. Further cerebellar titers were lower in comparison to other strains.

For variant B, the phenotype mostly resembled that of variant A with defined differences, including more elevated cerebellar bacterial titers. Elevated VEGF levels were found in animals infected with this allele. Vascular endothelial growth factor VEGF is secreted in the process of efferocytosis (Golpon et al., 2004; Vandivier et al., 2006). Elevated VEGF levels in the cerebrospinal fluid in cases of listeria meningitis is shown to be associated with poor outcome (Koopmans et al., 2014). However, this was not observed in our experimental model, neither on mortality rates nor in the level of brain damage.

Variant C presented with the worst clinical disease phenotype at 24 hpi and the most pronounced hippocampal damage assessed by quantification of apoptotic cells in the dentate gyrus of the hippocampus. Further variant C had significantly higher cerebellum titers compared to variant A. For variant D, we describe the most pronounced weight loss of all the variants and the highest bacterial titer in the cerebellum.

These observed differences when animals were infected with single variants demonstrate that changes in diseases severity are caused by the phase variable epigenetic modifications. These findings are in accordance to those observed by Manso et al. (2014) where RMS mutants showed differences in virulence in an intravenous mouse model of infection with *S. pneumoniae*. To permit a more detailed investigation into the interaction of bacteria expressing different *hsdS* alleles, we conducted competition experiments where either two or four *hsdS* variants were combined to create an inoculum.

Lees et al. (2017) were unable to detect observable selective advantages of *hsdS* variants in clinical blood and CSF samples, however analysis of these samples was complicated by the lack of known infecting strain. In contrast, a model infection as presented here, and in De Ste Croix et al. (2020b), with a starting inoculum of defined bacterial composition, allows for results to be analyzed in comparison to the inoculating dose and can therefore account for differences in the input population.

In PV RM systems there is likely to be continuous, stochastic variation at the loci. This results in a continuous re-generation of diversity within the bacterial population (De Ste Croix et al., 2020a). When a bacterial species enters a host there is a high probability of bottlenecking i.e., a significant reduction within the population is observed. Non-selective bottlenecks will frequently lead to high levels of divergence within the bacterial population of biological replicates, while an *in vivo* fitness advantage is indicated by a population continuously driven in a single direction (Aidley et al., 2017). The small but subtle differences we have observed in *hsdS* profiles when animals are infected with mixed inoculums suggests a potential competitive advantage. It should also be considered that strains which induce a greater level of damage may be selected against, and as a result be underrepresented within animals which survive until the end point of the experiment.

As there is little recombination of the RMS loci at higher temperatures (>30°C), we conducted mixed competition studies of different allelic combinations to look for selective advantages. When animals were infected with a mixture of 4 allelic variants, we did not identify any variant as having a selective advantage in either the CSF or the brain. To further explore the competition between populations expressing alternate *hsdS* alleles we infected animals with inoculums containing only two variants. This approach allows us to have a larger starting proportion to analyze against. Across multiple experiments we have observed a reduction in the proportion of the variant A when compared to the starting inoculum, in agreement with the clinical observations in our single variant experiments where animals infected with the variant A showed significantly better clinical scores at all time points. The loss of the variant A is accompanied by an increase in the variant C, further confirming the clinical score data of the single variant infections.

In the case of the infections with the inoculum composed of the 4 alleles, we designed 2 experimental groups that received antibiotic treatment or only saline, to determine whether antibiotic treatment could reveal a further selective advantage of one of the alleles. Such an advantage was not detected. However, we observed several differences between the two treatment groups. AMX treatment improve clinical parameters and decreased bacterial titers in the cerebellum. Survival was lower in saline-treated animals, without reaching statistical significance. In both groups, the observed mortality was in the range (0–25%) from what has been reported for neonates affected with neurolisteriosis (Mclauchlin, 1990; Mylonakis et al., 2002; Koopmans et al., 2017). Hippocampal apoptosis was also reduced. In contrast, treatment with amoxicillin worsens the size of the hydrocephalus. In a clinical setting, hydrocephali occur in 28% percent of neonatal cases (Hsieh et al., 2009). Bacterial lysis under antibiotic treatment could lead to an elevated release of bacterial components and therefore aggravated inflammation, as it is proposed for pneumococcal meningitis (Agyeman et al., 2014). However, in the present model, AMX treatment lead to a diminished expression of all analyzed pro-inflammatory and anti-inflammatory cytokines, so that inflammation couldn't account for the observed finding of increased hydrocephalus in AMX-treated animals.

Our model has certain limitations and displays differences when compared to human disease. For example, we did not find any pathognomic abscesses (Engelen-Lee et al., 2018) in the histological analysis, however, hydrocephalus occurred in all animals. Further, we use Wistar rats which express a wild type murine E-Cadherin which has been shown not to interact with the cell invasion factor internalin InlA (Lecuit et al., 1999, 2001). Our model does bypass the different physiological barriers the bacteria must cross in a natural food-borne infection by injecting the pathogen into the cisterna magna. In doing so, we have consistent CNS infection, but we remove the initial step of bacterial invasion. In this model, we therefore couldn't investigate on the role of this phase variable loci early in the disease process, but only when CNS invasion has been established.

This study allowed us to gain a better insight on bacterial determinants that influence virulence in neonates affected by listeria meningitis, in particular the development of brain damage. This will be important to understand how the damage translates into neurofunctional deficits, in order to develop specific therapeutic strategies.

Work is on-going to fully understand the phenotypic attributes of this PV RMS of *Listeria monocytogenes* CC4 serotype 4b, however we observe differences in virulence in an acute meningitis/meningoencephalitis model. We now shall investigate long-term outcomes and further look into the biochemical mechanics of this altered virulence.

## Data Availability Statement

The original contributions presented in the study are included in the article/Supplementary Material, further inquiries can be directed to the corresponding author/s.

## Ethics Statement

The animal study was reviewed and approved by the Animal Care and Experimentation Committee of the Canton of Bern, Switzerland (license no. BE 01/18).

## Author Contributions

FZ designed the studies, performed animal experiments, analyzed the results, and contributed to manuscript preparation. DG designed the study, analyzed the results, co-supervised the studies, and contributed to manuscript preparation. SL designed the study, co-supervised the studies, and contributed to manuscript preparation. MD conducted GeneScan analysis of the inoculum, CSF and cerebellum samples, conducted statistical analysis, and contributed to the manuscript. RH conducted WGS of Listeria strains and developed the GeneScan protocol. RZ analyzed WGS data. IV isolated *hsdS* strains and developed the GeneScan protocol. EG isolated *hsdS* strains. MO analyzed WGS data, provided supervision, and contributed to the manuscript. All authors contributed to the article and approved the submitted version.

## Conflict of Interest

The authors declare that the research was conducted in the absence of any commercial or financial relationships that could be construed as a potential conflict of interest.

## References

[B1] Agyeman P. Grandgirard D. Leib S. L. (2014). Pathogenesis and pathophysiology of bacterial infections, in Infections of the Central Nervous System, 4th Edn, eds Scheld M. W. Whitley R. J. Marra C. M. (Philadelphia, PA: Lippincott Williams & Wilkins).

[B2] Aidley J. Rajopadhye S. Akinyemi N. M. Lango-Scholey L. Jones M. A. Bayliss C. D. (2017). Nonselective bottlenecks control the divergence and diversification of phase-variable bacterial populations. mBio 8:16 10.1128/mBio.02311-16 PMC5380846 28377533

[B3] Althaus D. Lehner A. Brisse S. Maury M. Tasara T. Stephan R. (2014). Characterization of Listeria monocytogenes strains isolated during 2011-2013 from human infections in Switzerland. Foodborne Pathog. Dis. 11, 753–758. 10.1089/fpd.2014.1747 25007293

[B4] Atack J. M. Srikhanta Y. N. Fox K. L. Jurcisek J. A. Brockman K. L. Clark T. A.. (2015). A biphasic epigenetic switch controls immunoevasion, virulence and niche adaptation in non-typeable Haemophilus influenzae. Nat. Commun. 6:7828. 10.1038/ncomms8828 26215614 PMC4525171

[B5] Atack J. M. Weinert L. A. Tucker A. W. Husna A. U. Wileman T. M. Hadjirin N. F.. (2018). Streptococcus suis contains multiple phase-variable methyltransferases that show a discrete lineage distribution. Nucleic Acids Res. 46, 11466–11476. 10.1093/nar/gky913 30304532 PMC6265453

[B6] Bedford H. De Louvois J. Halket S. Peckham C. Hurley R. Harvey D. (2001). Meningitis in infancy in England and Wales: follow up at age 5 years. BMJ 323, 533–536. 10.1136/bmj.323.7312.533 11546697 PMC48156

[B7] Bifrare Y. D. Gianinazzi C. Imboden H. Leib S. L. Täuber M. G. (2003). Bacterial meningitis causes two distinct forms of cellular damage in the hippocampal dentate gyrus in infant rats. Hippocampus 13, 481–488. 10.1002/hipo.10142 12836917

[B8] Charlier C. Perrodeau E. Leclercq A. Cazenave B. Pilmis B. Henry B.. (2017). Clinical features and prognostic factors of listeriosis: the MONALISA national prospective cohort study. Lancet Infect. Dis. 17, 510–519. 10.1016/S1473-3099(16)30521-7 28139432

[B9] Chen P. Den Bakker H. C. Korlach J. Kong N. Storey D. B. Paxinos E. E. (2017). Comparative genomics reveals the diversity of restriction-modification systems and dna methylation sites in listeria monocytogenes. Appl. Environ. Microbiol. 83, e02091–16. 10.1128/AEM.02091-16 27836852 PMC5244299

[B10] Connor T. R. Loman N. J. Thompson S. Smith A. Southgate J. Poplawski R.. (2016). CLIMB (the Cloud Infrastructure for Microbial Bioinformatics): an online resource for the medical microbiology community. Microb. Genom. 2:e000086. 10.1099/mgen.0.000086 28785418 PMC5537631

[B11] de Noordhout C. M. Devleesschauwer B. Angulo F. J. Verbeke G. Haagsma J. Kirk M.. (2014). The global burden of listeriosis: a systematic review and meta-analysis. Lancet Infect. Dis. 14, 1073–1082. 10.1016/S1473-3099(14)70870-9 25241232 PMC4369580

[B12] De Ste Croix M. Holmes J. Wanford J. J. Moxon E. R. Oggioni M. R. Bayliss C. D. (2020a). Selective and non-selective bottlenecks as drivers of the evolution of hypermutable bacterial loci. Mol. Microbiol. 113, 672–681. 10.1111/mmi.14453 32185830 PMC7154626

[B13] De Ste Croix M. Mitsi E. Morozov A. Glenn S. Andrew P. W. Ferreira D. M.. (2020b). Phase variation in pneumococcal populations during carriage in the human nasopharynx. Sci. Rep. 10:1803. 10.1038/s41598-020-58684-2 32019989 PMC7000782

[B14] De Ste Croix M. Vacca I. Kwun M. J. Ralph J. D. Bentley S. D. Haigh R.. (2017). Phase-variable methylation and epigenetic regulation by type I restriction-modification systems. FEMS Microbiol. Rev. 41, S3–S15. 10.1093/femsre/fux025 28830092

[B15] De Ste Croix M. Chen K. Y. Vacca I. Manso A. S. Johnston C. Polard P.. (2019). Recombination of the phase-variable spnIII locus is independent of all known pneumococcal site-specific recombinases. J. Bacteriol. 201:e00233–00219. 10.1128/JB.00233-19 31085693 PMC6620402

[B16] Disson O. Lecuit M. (2012). Targeting of the central nervous system by Listeria monocytogenes. Virulence 3, 213–221. 10.4161/viru.19586 22460636 PMC3396700

[B17] Engelen-Lee J. Y. Koopmans M. M. Brouwer M. C. Aronica E. Van De Beek D. (2018). Histopathology of listeria meningitis. J. Neuropathol. Exp. Neurol. 77, 950–957. 10.1093/jnen/nly077 30169667 PMC6140438

[B18] Fagerlund A. Langsrud S. Schirmer B. C. Moretro T. Heir E. (2016). Genome analysis of listeria monocytogenes sequence type 8 strains persisting in salmon and poultry processing environments and comparison with related strains. PLoS ONE 11:e0151117. 10.1371/journal.pone.0151117 26953695 PMC4783014

[B19] Golpon H. A. Fadok V. A. Taraseviciene-Stewart L. Scerbavicius R. Sauer C. Welte T.. (2004). Life after corpse engulfment: phagocytosis of apoptotic cells leads to VEGF secretion and cell growth. FASEB J. 18, 1716–1718. 10.1096/fj.04-1853fje 15345697

[B20] Grandgirard D. Schurch C. Cottagnoud P. Leib S. L. (2007a). Prevention of brain injury by the nonbacteriolytic antibiotic daptomycin in experimental pneumococcal meningitis. Antimicrob Agents Chemother 51, 2173–2178. 10.1128/AAC.01014-06 17371820 PMC1891377

[B21] Grandgirard D. Steiner O. Täuber M. G. Leib S. L. (2007b). An infant mouse model of brain damage in pneumococcal meningitis. Acta Neuropathol. 114, 609–617. 10.1007/s00401-007-0304-8 17938941

[B22] Hsieh W. S. Tsai L. Y. Jeng S. F. Hsu C. H. Lin H. C. Hsueh P. R.. (2009). Neonatal listeriosis in Taiwan, 1990-2007. Int. J. Infect. Dis. 113, 193–195. 10.1016/j.ijid.2008.06.006 18768340

[B23] Kasanmoentalib E. S. Brouwer M. C. Van Der Ende A. Van De Beek D. (2010). Hydrocephalus in adults with community-acquired bacterial meningitis. Neurology 75, 918–923. 10.1212/WNL.0b013e3181f11e10 20820003

[B24] Koopmans M. M. Bijlsma M. W. Brouwer M. C. Van De Beek D. Van Der Ende A. (2017). Listeria monocytogenes meningitis in the Netherlands, 1985-2014: a nationwide surveillance study. J. Infect. 75, 12–19. 10.1016/j.jinf.2017.04.004 28419853 PMC5513958

[B25] Koopmans M. M. Brouwer M. C. Bijlsma M. W. Bovenkerk S. Keijzers W. Van Der Ende A.. (2013). Listeria monocytogenes sequence type 6 and increased rate of unfavorable outcome in meningitis: epidemiologic cohort study. Clin. Infect. Dis. 57, 247–253. 10.1093/cid/cit250 23592828

[B26] Koopmans M. M. Brouwer M. C. Geldhoff M. Seron M. V. Houben J. Van Der Ende A.. (2014). Cerebrospinal fluid inflammatory markers in patients with Listeria monocytogenes meningitis. BBA Clin. 1, 44–51. 10.1016/j.bbacli.2014.06.001 25960946 PMC4418767

[B27] Koopmans M. M. Engelen-Lee J. Brouwer M. C. Jaspers V. Man W. K. Vall Seron M.. (2018). Characterization of a Listeria monocytogenes meningitis mouse model. J. Neuroinflammation. 15:257. 10.1186/s12974-018-1293-3 30193592 PMC6128981

[B28] Kwun M. J. Oggioni M. R. De Ste Croix M. Bentley S. D. Croucher N. J. (2018). Excision-reintegration at a pneumococcal phase-variable restriction-modification locus drives within- and between-strain epigenetic differentiation and inhibits gene acquisition. Nucleic Acids Res. 46, 11438–11453. 10.1093/nar/gky906 30321375 PMC6265443

[B29] Lecuit M. Dramsi S. Gottardi C. Fedor-Chaiken M. Gumbiner B. Cossart P. (1999). A single amino acid in E-cadherin responsible for host specificity towards the human pathogen Listeria monocytogenes. EMBO J. 18, 3956–3963. 10.1093/emboj/18.14.3956 10406800 PMC1171471

[B30] Lecuit M. Vandormael-Pournin S. Lefort J. Huerre M. Gounon P. Dupuy C.. (2001). A transgenic model for listeriosis: role of internalin in crossing the intestinal barrier. Science 292, 1722–1725. 10.1126/science.1059852 11387478

[B31] Lee J. Y. H. Carter G. P. Pidot S. J. Guerillot R. Seemann T. Goncalves Da Silva A.. (2019). mining the methylome reveals extensive diversity in staphylococcus epidermidis restriction modification. mBio 10:19. 10.1128/mBio.02451-19 31848274 PMC6918075

[B32] Lees J. A. Kremer P. H. C. Manso A. S. Croucher N. J. Ferwerda B. Seron M. V. (2017). Large scale genomic analysis shows no evidence for pathogen adaptation between the blood and cerebrospinal fluid niches during bacterial meningitis. Microb. Genom. 3:e000103 10.1099/mgen.0.000103 28348877 PMC5361624

[B33] Leib S. L. Heimgartner C. Bifrare Y. D. Loeffler J. M. Täuber M. G. (2003). Dexamethasone aggravates hippocampal apoptosis and learning deficiency in pneumococcal meningitis in infant rats. Pediatr. Res. 54, 353–357. 10.1203/01.PDR.0000079185.67878.72 12788989

[B34] Leib S. L. Kim Y. S. Chow L. L. Sheldon R. A. Täuber M. G. (1996). Reactive oxygen intermediates contribute to necrotic and apoptotic neuronal injury in an infant rat model of bacterial meningitis due to group B streptococci. J. Clin. Invest. 98, 2632–2639. 10.1172/JCI119084 8958228 PMC507723

[B35] Li J. Li J. W. Feng Z. Wang J. An H. Liu Y.. (2016). Epigenetic Switch Driven by DNA Inversions Dictates Phase Variation in Streptococcus pneumoniae. PLoS Pathog. 12:e1005762. 10.1371/journal.ppat.1005762 27427949 PMC4948785

[B36] Loeffler J. M. Ringer R. Hablutzel M. Täuber M. G. Leib S. L. (2001). The free radical scavenger alpha-phenyl-tert-butyl nitrone aggravates hippocampal apoptosis and learning deficits in experimental pneumococcal meningitis. J. Infect. Dis. 183, 247–252. 10.1086/317921 11110643

[B37] Manso A. S. Chai M. H. Atack J. M. Furi L. De Ste Croix M. Haigh R.. (2014). A random six-phase switch regulates pneumococcal virulence via global epigenetic changes. Nat. Commun. 5:5055. 10.1038/ncomms6055 25268848 PMC4190663

[B38] Mclauchlin J. (1990). Human listeriosis in Britain, 1967-85, a summary of 722 cases. 1. Listeriosis during pregnancy and in the newborn. Epidemiol Infect 104, 181–189. 10.1017/S0950268800059343 2108869 PMC2271760

[B39] Michelet C. Leib S. L. Bentue-Ferrer D. Täuber M. G. (1999). Comparative efficacies of antibiotics in a rat model of meningoencephalitis due to *Listeria monocytogenes*. Antimicrob Agents Chemother. 43, 1651–1656. 10.1128/AAC.43.7.1651 10390217 PMC89338

[B40] Muri L. Grandgirard D. Buri M. Perny M. Leib S. L. (2018). Combined effect of non-bacteriolytic antibiotic and inhibition of matrix metalloproteinases prevents brain injury and preserves learning, memory and hearing function in experimental paediatric pneumococcal meningitis. J. Neuroinflam. 15:233. 10.1186/s12974-018-1272-8 30131074 PMC6103863

[B41] Mylonakis E. Paliou M. Hohmann E. L. Calderwood S. B. Wing E. J. (2002). Listeriosis during pregnancy: a case series and review of 222 cases. Medicine 81, 260–269. 10.1097/00005792-200207000-00002 12169881

[B42] Nau R. Soto A. Bruck W. (1999). Apoptosis of neurons in the dentate gyrus in humans suffering from bacterial meningitis. J. Neuropathol. Exp. Neurol. 58, 265–274. 10.1097/00005072-199903000-00006 10197818

[B43] Pagliano P. Arslan F. Ascione T. (2017). Epidemiology and treatment of the commonest form of listeriosis: meningitis and bacteraemia. Infez. Med. 25, 210–216. 28956537

[B44] Pelegrin I. Moragas M. Suarez C. Ribera A. Verdaguer R. Martinez-Yelamos S.. (2014). Listeria monocytogenes meningoencephalitis in adults: analysis of factors related to unfavourable outcome. Infection 42, 817–827. 10.1007/s15010-014-0636-y 24902522

[B45] Perny M. Roccio M. Grandgirard D. Solyga M. Senn P. Leib S. L. (2016). The severity of infection determines the localization of damage and extent of sensorineural hearing loss in experimental pneumococcal meningitis. J. Neurosci. 36, 7740–7749. 10.1523/JNEUROSCI.0554-16.2016 27445150 PMC6705551

[B46] Remer K. A. Jungi T. W. Fatzer R. Täuber M. G. Leib S. L. (2001). Nitric oxide is protective in listeric meningoencephalitis of rats. Infect. Immun. 69, 4086–4093. 10.1128/IAI.69.6.4086-4093.2001 11349080 PMC98473

[B47] Semple B. D. Blomgren K. Gimlin K. Ferriero D. M. Noble-Haeusslein L. J. (2013). Brain development in rodents and humans: Identifying benchmarks of maturation and vulnerability to injury across species. Prog. Neurobiol. 106–107, 1–16. 10.1016/j.pneurobio.2013.04.001 23583307 PMC3737272

[B48] Sitaraman R. Denison A. M. Dybvig K. (2002). A unique, bifunctional site-specific DNA recombinase from Mycoplasma pulmonis. Mol. Microbiol. 46, 1033–1040. 10.1046/j.1365-2958.2002.03206.x 12421309

[B49] Srikhanta Y. N. Maguire T. L. Stacey K. J. Grimmond S. M. Jennings M. P. (2005). The phasevarion: a genetic system controlling coordinated, random switching of expression of multiple genes. Proc. Natl. Acad. Sci. U.S.A. 102, 5547–5551. 10.1073/pnas.0501169102 15802471 PMC556257

[B50] Tunkel A. R. Hartman B. J. Kaplan S. L. Kaufman B. A. Roos K. L. Scheld W. M.. (2004). Practice guidelines for the management of bacterial meningitis. Clin. Infect. Dis. 39, 1267–1284. 10.1086/425368 15494903

[B51] van de Beek D. Cabellos C. Dzupova O. Esposito S. Klein M. Kloek A. T.. (2016). ESCMID guideline: diagnosis and treatment of acute bacterial meningitis. Clin. Microbiol. Infect. 22(Suppl 3), S37–S62. 10.1016/j.cmi.2016.01.007 27062097

[B52] Vandivier R. W. Henson P. M. Douglas I. S. (2006). Burying the dead: the impact of failed apoptotic cell removal (efferocytosis) on chronic inflammatory lung disease. Chest 129, 1673–1682. 10.1378/chest.129.6.1673 16778289

[B53] Wellmer A. Noeske C. Gerber J. Munzel U. Nau R. (2000). Spatial memory and learning deficits after experimental pneumococcal meningitis in mice. Neurosci. Lett. 296, 137–140. 10.1016/S0304-3940(00)01645-1 11109000

[B54] Yildiz O. Aygen B. Esel D. Kayabas U. Alp E. Sumerkan B.. (2007). Sepsis and meningitis due to Listeria monocytogenes. Yonsei. Med. J. 48, 433–439. 10.3349/ymj.2007.48.3.433 17594151 PMC2628085

[B55] Yu G. C. Smith D. K. Zhu H. C. Guan Y. Lam T. T. Y. (2017). GGTREE: an R package for visualization and annotation of phylogenetic trees with their covariates and other associated data. Methods Ecol. Evol. 8, 28–36. 10.1111/2041-210X.12628

[B56] Zamudio R. Haigh R. D. Ralph J. D. De Ste Croix M. Tasara T. Zurfluh K.. (2020). Lineage-specific evolution and gene flow in Listeria monocytogenes are independent of bacteriophages. Environ. Microbiol. 10.1111/1462-2920.15111. [Epub ahead of print]. 32483914 PMC7614921

