# Supplementary material for: Pathogenic Differences of Type 1 Restriction-Modification Allele Variants in Experimental Listeria monocytogenes Meningitis
Source: Front Cell Infect Microbiol. 2020 Oct 30;10:590657. doi: 10.3389/fcimb.2020.590657 (PMC7662400; doi:10.3389/fcimb.2020.590657)
Supplement: Supplementary file 1 [file Image_1.PDF]

Supplementary Figure 1

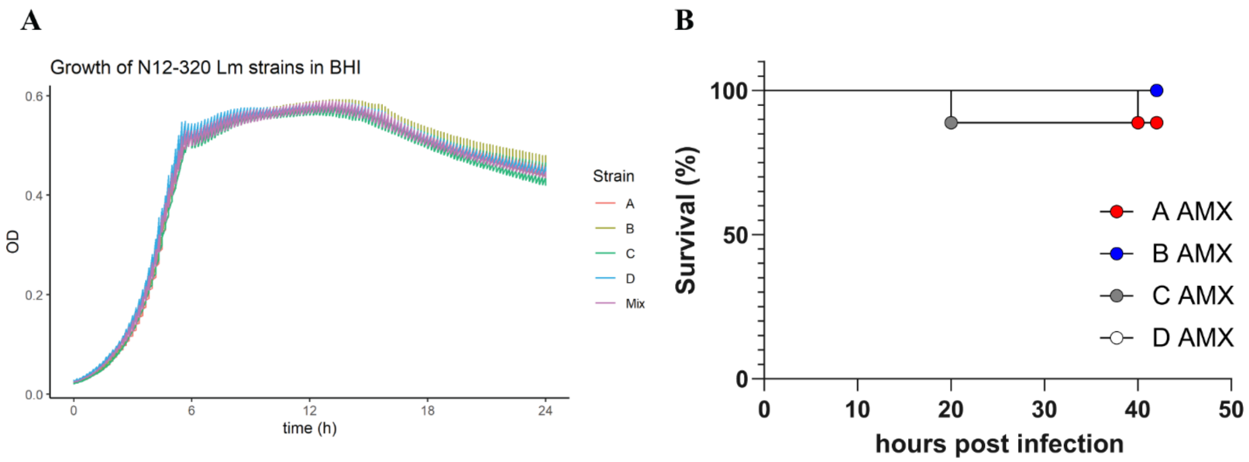

Supplementary Figure 1: Effect of the alleles on *in vitro* growth and survival of the animals *in vivo*: The different alleles displayed similar growth rate (A). Survival was not significantly altered by the infection with a different RMS allele (B) (A:n=9, B:n= 8, C:n= 9, D:n = 8).
