# Supplementary material for: Pathogenic Differences of Type 1 Restriction-Modification Allele Variants in Experimental Listeria monocytogenes Meningitis
Source: Front Cell Infect Microbiol. 2020 Oct 30;10:590657. doi: 10.3389/fcimb.2020.590657 (PMC7662400; doi:10.3389/fcimb.2020.590657)
Supplement: Supplementary file 2 [file Image_2.PDF]

Supplementary Figure 2

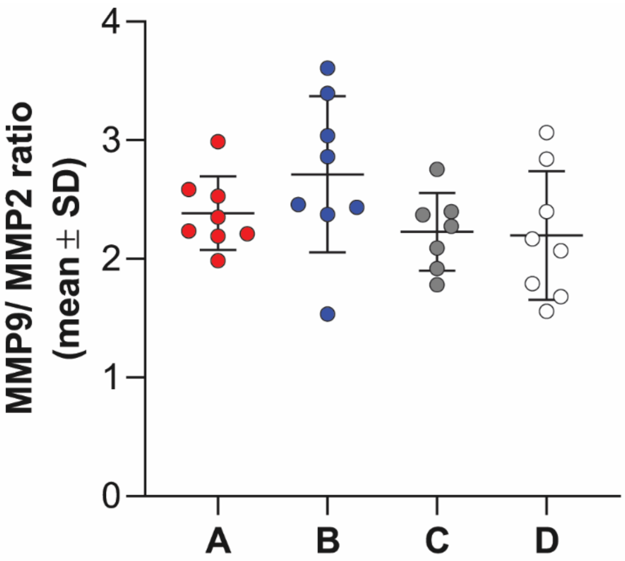

Supplementary Figure 2: Matrix-metalloproteinases in infection with single RMS allele variant: Matrix metalloproteinase-9 (MMP9) expression normalized to the basally expressed MMP2 measured in cerebellum homogenate at 42hpi (A:n=8, B:n= 8, C:n= 8, D:n = 8).. No difference between the variants found (one-way ANOVA p= 0.1627)
