# Supplementary material for: Pathogenic Differences of Type 1 Restriction-Modification Allele Variants in Experimental Listeria monocytogenes Meningitis
Source: Front Cell Infect Microbiol. 2020 Oct 30;10:590657. doi: 10.3389/fcimb.2020.590657 (PMC7662400; doi:10.3389/fcimb.2020.590657)
Supplement: Supplementary file 3 [file Image_3.PDF]

Supplementary Figure 3

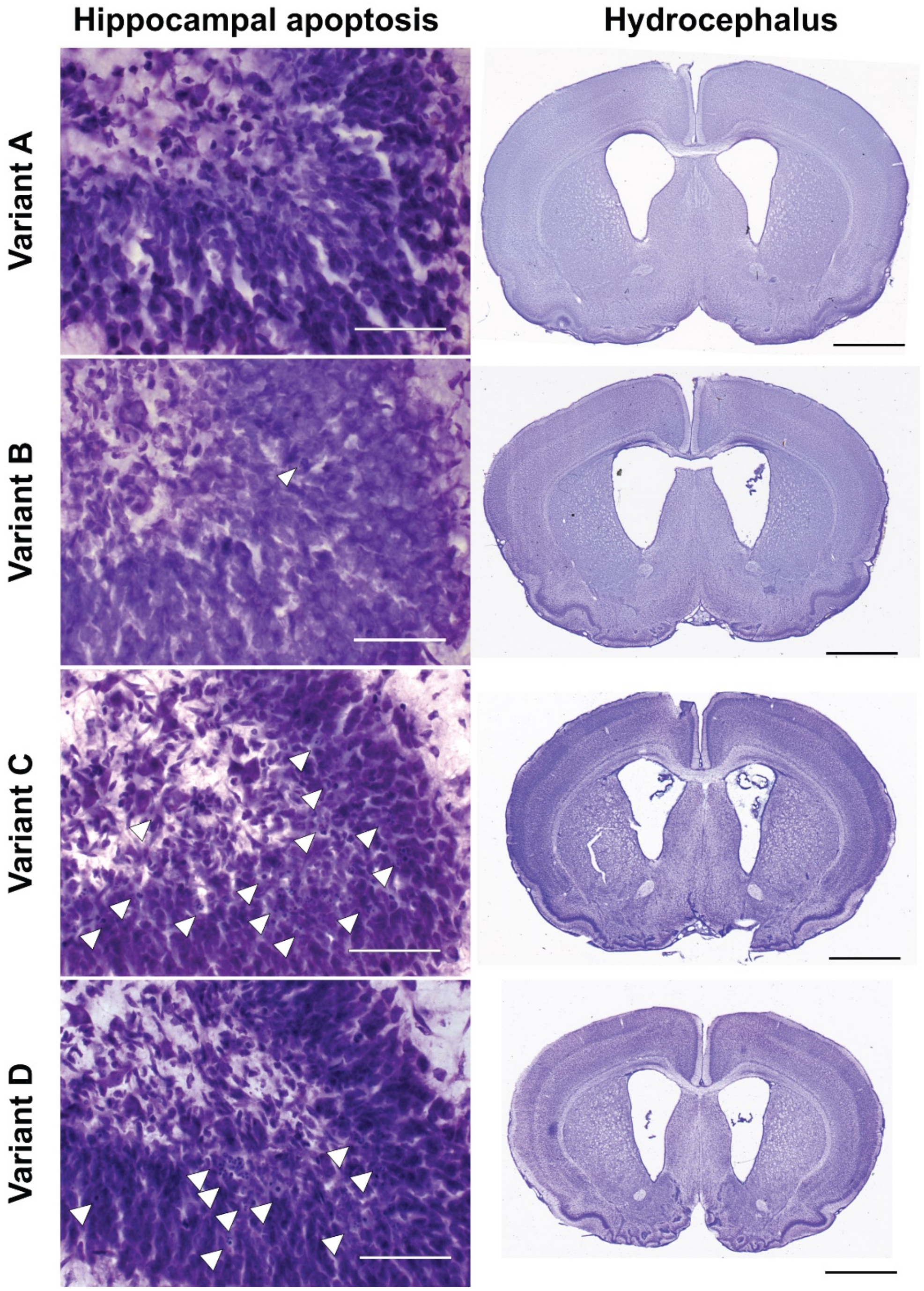

Supplementary Figure 3: Representative pictures of cresyl violet-stained sections of the apex of the dentate gyrus (left pictures, bar size 50 µm), with examples of apoptotic cells indicated with white arrowheads, or from entire sections with enlarged lateral ventricles (right pictures, bar size 2 mm) of animals infected with the four different variants.
