# Supplementary material for: Pathogenic Differences of Type 1 Restriction-Modification Allele Variants in Experimental Listeria monocytogenes Meningitis
Source: Front Cell Infect Microbiol. 2020 Oct 30;10:590657. doi: 10.3389/fcimb.2020.590657 (PMC7662400; doi:10.3389/fcimb.2020.590657)
Supplement: Supplementary file 4 [file Image_4.pdf]

Supplementary Figure 4

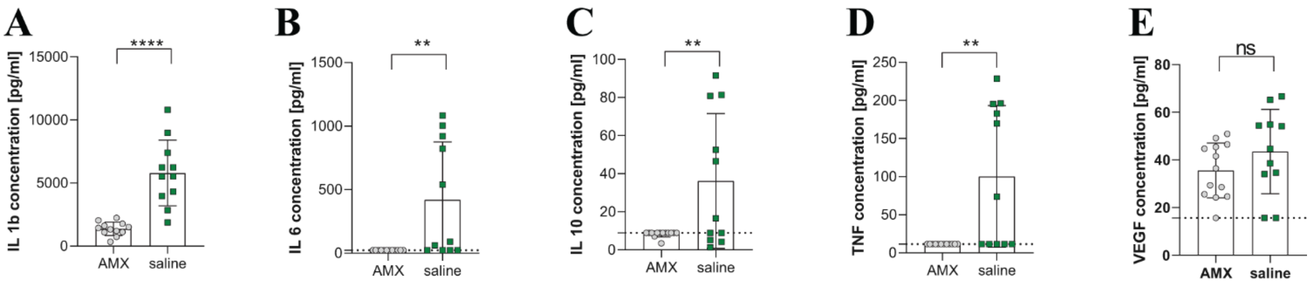

Supplementary Figure 4: Inflammatory parameters in infection with a mixture of the 4 RMS allele variants (A:B:C:D): Cytokine levels measured in cerebellum homogenate at 42hpi. (AMX= 13, control= 11) show an elevated pro-inflammatory (IL-1β, IL-6, TNF-α, VEGF) and anti-inflammatory (IL-10) cytokine profile. No significance reportable for VEGF-levels. The dotted line represents the lower limit of detection (LOD) for each cytokine measured. Data point on the line represent samples with levels under the detection limits, for which the corresponding value of the limit of detection has been substituted to allow statistical analysis.
