# Supplementary material for: Pathogenic Differences of Type 1 Restriction-Modification Allele Variants in Experimental Listeria monocytogenes Meningitis
Source: Front Cell Infect Microbiol. 2020 Oct 30;10:590657. doi: 10.3389/fcimb.2020.590657 (PMC7662400; doi:10.3389/fcimb.2020.590657)
Supplement: Supplementary file 5 [file Data_Sheet_1.PDF]

Supplementary Material

1 Supplementary table 1: Summary table of all analysed parameters for infections experiments with single RMS variant or the mixture of 4 RMS variants.

		**single RMS variant**						**4 RMS variants mix**
		**A vs. B**	**A vs. C**	**A vs. D**	**B vs. C**	**B vs. D**	**C vs. D**	**AMX treated vs. control**
**clinical score**	18hpi	ns	A > C ****	A < D ***	B > C ***	B > D **	ns	N/A
	24hpi	ns	A > C *	ns	ns	ns	ns	N/A
	42hpi	ns	A > C ***	ns	B > C **	ns	ns	AMX > control *
**weight change**	18hpi	ns	ns	A < D *	ns	B < D **	ns	N/A
	24hpi	ns	ns	A < D **	ns	B < D **	ns	N/A
	42hpi	ns	ns	A < D *	ns	ns	ns	ns
**cerebellum bacterial titer 42hpi**		A < B *	A < C **	A < D ****	ns	ns	ns	AMX > control ****
**CSF bacterial titer 24hpi**		ns	ns	ns	ns	ns	ns	N/A
**IL 1 concentration**		ns	ns	A < D *	ns	ns	ns	AMX > control ****
**IL6 concentration**		N/A	N/A	N/A	N/A	N/A	N/A	AMX > control **
**IL10 concentration**		N/A	N/A	N/A	N/A	N/A	N/A	AMX > control **
**IL18 concentration**		ns	ns	ns	ns	ns	ns	N/A
**VEGFconcentration**		A > B **	A > C *	ns	B > C ****	B > D ****	ns	ns
**TNF concentration**		N/A	N/A	N/A	N/A	N/A	N/A	AMX > control **
**mean hippocampal apoptosis count**		ns	A < C *	ns	ns	ns	ns	AMX > control *
**Volume of lateral and 3rd ventricle**		ns	ns	ns	ns	ns	ns	AMX < control *

Cells shown in green represent statistically significant results, in red non-significant. N/A: not assessed. **** p<0.0001, *** p<0.001, ** p<0.01, * p<0.05

2 Supplementary table 2: Detailed statistical analysis of difference in clinical scores in animals infected with single allelic variants

Tukey's multiple comparisons test	Predicted (LS) mean diff.	95.00% CI of diff.	Significant?	Summary	Adjusted P Value
18					
A AMX vs. B AMX	0.05556	-0.3724 to 0.4835	No	ns	0.9866
A AMX vs. C AMX	0.7778	0.3499 to 1.206	Yes	****	<0.0001
A AMX vs. D AMX	0.6667	0.2388 to 1.095	Yes	***	0.0005
B AMX vs. C AMX	0.7222	0.2943 to 1.150	Yes	***	0.0001
B AMX vs. D AMX	0.6111	0.1832 to 1.039	Yes	**	0.0017
C AMX vs. D AMX	-0.1111	-0.5390 to 0.3168	No	ns	0.9059
24					
A AMX vs. B AMX	0.1111	-0.3168 to 0.5390	No	ns	0.9059
A AMX vs. C AMX	0.4832	0.04306 to 0.9233	Yes	*	0.0254
A AMX vs. D AMX	0.3889	-0.03902 to 0.8168	No	ns	0.0890
B AMX vs. C AMX	0.3721	-0.06805 to 0.8122	No	ns	0.1285
B AMX vs. D AMX	0.2778	-0.1501 to 0.7057	No	ns	0.3330
C AMX vs. D AMX	-0.09429	-0.5344 to 0.3458	No	ns	0.9442
42					
A AMX vs. B AMX	0.1219	-0.3296 to 0.5734	No	ns	0.8956
A AMX vs. C AMX	0.7610	0.3092 to 1.213	Yes	***	0.0001
A AMX vs. D AMX	0.4365	-0.01500 to 0.8879	No	ns	0.0621
B AMX vs. C AMX	0.6391	0.1873 to 1.091	Yes	**	0.0019
B AMX vs. D AMX	0.3146	-0.1369 to 0.7660	No	ns	0.2714
C AMX vs. D AMX	-0.3245	-0.7762 to 0.1272	No	ns	0.2460

3 **Supplementary table 3: Detailed statistical analysis of difference in weight in animals infected with single allelic variants.**

Tukey's multiple comparisons test	Mean Diff.	95.00% CI of diff.	Significant?	Summary	Adjusted P Value
18					
A AMX vs. B AMX	-0.4711	-3.478 to 2.536	No	ns	0.969
A AMX vs. C AMX	2.979	-0.9305 to 6.888	No	ns	0.1669
A AMX vs. D AMX	3.349	0.7074 to 5.990	Yes	*	0.0115
B AMX vs. C AMX	3.45	-0.4485 to 7.348	No	ns	0.0911
B AMX vs. D AMX	3.82	1.202 to 6.438	Yes	**	0.0039
C AMX vs. D AMX	0.37	-3.324 to 4.064	No	ns	0.9901
24					
A AMX vs. B AMX	-0.2089	-3.287 to 2.869	No	ns	0.9973
A AMX vs. C AMX	3.226	-0.7208 to 7.173	No	ns	0.124
A AMX vs. D AMX	4.001	1.350 to 6.652	Yes	**	0.0027
B AMX vs. C AMX	3.435	-0.6632 to 7.533	No	ns	0.1142
B AMX vs. D AMX	4.21	1.246 to 7.174	Yes	**	0.0048
C AMX vs. D AMX	0.7749	-3.108 to 4.658	No	ns	0.9295
42					
A AMX vs. B AMX	2.143	-4.768 to 9.053	No	ns	0.8023
A AMX vs. C AMX	6.099	-1.375 to 13.57	No	ns	0.1269
A AMX vs. D AMX	7.094	1.423 to 12.76	Yes	*	0.013
B AMX vs. C AMX	3.956	-4.031 to 11.94	No	ns	0.4959
B AMX vs. D AMX	4.951	-1.554 to 11.46	No	ns	0.1632
C AMX vs. D AMX	0.995	-6.141 to 8.131	No	ns	0.9744
